# Virological characteristics of a SARS-CoV-2-related bat coronavirus, BANAL-20-236

**DOI:** 10.1016/j.ebiom.2024.105181

**Published:** 2024-06-04

**Authors:** Shigeru Fujita, Arnon Plianchaisuk, Sayaka Deguchi, Hayato Ito, Naganori Nao, Lei Wang, Hesham Nasser, Tomokazu Tamura, Izumi Kimura, Yukie Kashima, Rigel Suzuki, Saori Suzuki, Izumi Kida, Masumi Tsuda, Yoshitaka Oda, Rina Hashimoto, Yukio Watanabe, Keiya Uriu, Daichi Yamasoba, Ziyi Guo, Alfredo A. Hinay, Yusuke Kosugi, Luo Chen, Lin Pan, Yu Kaku, Hin Chu, Flora Donati, Sarah Temmam, Marc Eloit, Yuki Yamamoto, Tetsuharu Nagamoto, Hiroyuki Asakura, Mami Nagashima, Kenji Sadamasu, Kazuhisa Yoshimura, Yutaka Suzuki, The Genotype to Phenotype Japan (G2P-Japan) Consortium, Hirofumi Sawa, Keita Mizuma, Jingshu Li, Yume Mimura, Yuma Ohari, Tomoya Tsubo, Zannatul Ferdous, Kenji Shishido, Hiromi Mohri, Miki Iida, Shuhei Tsujino, Naoko Misawa, Kaoru Usui, Wilaiporn Saikruang, Spyridon Lytras, Shusuke Kawakubo, Luca Nishumura, Jarel Elgin Mendoza Tolentino, Wenye Li, Maximilian Stanley Yo, Kio Horinaka, Mai Suganami, Mika Chiba, Ryo Yoshimura, Kyoko Yasuda, Keiko Iida, Adam Patrick Strange, Naomi Ohsumi, Shiho Tanaka, Eiko Ogawa, Kaho Okumura, Tsuki Fukuda, Rina Osujo, Isao Yoshida, So Nakagawa, Akifumi Takaori-Kondo, Kotaro Shirakawa, Kayoko Nagata, Ryosuke Nomura, Yoshihito Horisawa, Yusuke Tashiro, Yugo Kawai, Yoshitaka Nakata, Hiroki Futatsusako, Ayaka Sakamoto, Naoko Yasuhara, Takao Hashiguchi, Tateki Suzuki, Kanako Kimura, Jiei Sasaki, Yukari Nakajima, Hisano Yajima, Takashi Irie, Ryoko Kawabata, Kaori Sasaki-Tabata, Ryo Shimizu, M.S.T. Monira Begum, Michael Jonathan, Yuka Mugita, Sharee Leong, Otowa Takahashi, Kimiko Ichihara, Takamasa Ueno, Chihiro Motozono, Mako Toyoda, Akatsuki Saito, Anon Kosaka, Miki Kawano, Natsumi Matsubara, Tomoko Nishiuchi, Jiri Zahradnik, Prokopios Andrikopoulos, Miguel Padilla-Blanco, Aditi Konar, Jumpei Ito, Terumasa Ikeda, Shinya Tanaka, Keita Matsuno, Takasuke Fukuhara, Kazuo Takayama, Kei Sato

**Affiliations:** a Division of Systems Virology, Department of Microbiology and Immunology, The Institute of Medical Science, The University of Tokyo, Tokyo, Japan; b Graduate School of Medicine, The University of Tokyo, Tokyo, Japan; c Center for iPS Cell Research and Application (CiRA), Kyoto University, Kyoto, Japan; d Department of Microbiology and Immunology, Faculty of Medicine, Hokkaido University, Sapporo, Japan; e One Health Research Center, Hokkaido University, Sapporo, Japan; f Institute for Vaccine Research and Development (IVReD), Hokkaido University, Sapporo, Japan; g Division of International Research Promotion, International Institute for Zoonosis Control, Hokkaido University, Sapporo, Japan; h Department of Cancer Pathology, Faculty of Medicine, Hokkaido University, Sapporo, Japan; i Institute for Chemical Reaction Design and Discovery (WPI-ICReDD), Hokkaido University, Sapporo, Japan; j Division of Molecular Virology and Genetics, Joint Research Center for Human Retrovirus infection, Kumamoto University, Kumamoto, Japan; k Department of Clinical Pathology, Faculty of Medicine, Suez Canal University, Ismailia, Egypt; l Graduate School of Frontier Sciences, The University of Tokyo, Chiba, Japan; m Division of Risk Analysis and Management, International Institute for Zoonosis Control, Hokkaido University, Sapporo, Japan; n Faculty of Medicine, Kobe University, Kobe, Japan; o State Key Laboratory of Emerging Infectious Diseases, Department of Microbiology, School of Clinical Medicine, Li Ka Shing Faculty of Medicine, The University of Hong Kong, Pokfulam, Hong Kong Special Administrative Region, China; p Institut Pasteur, Université Paris Cité, CNRS UMR 3569, Molecular Genetics of RNA Viruses Unit, Paris, France; q Institut Pasteur, Université Paris Cité, National Reference Center for Respiratory Viruses, Paris, France; r Institut Pasteur, Université Paris Cité, Pathogen Discovery Laboratory, Paris, France; s Institut Pasteur, Université Paris Cité, The WOAH(OIE) Collaborating Center for the Detection and Identification in Humans of Emerging Animal Pathogens, Paris, France; t HiLung Inc., Kyoto, Japan; u Tokyo Metropolitan Institute of Public Health, Tokyo, Japan; v International Research Center for Infectious Diseases, The Institute of Medical Science, The University of Tokyo, Tokyo, Japan; w International Collaboration Unit, International Institute for Zoonosis Control, Hokkaido University, Sapporo, Japan; x AMED-CREST, Japan Agency for Medical Research and Development (AMED), Tokyo, Japan; y Laboratory of Virus Control, Research Institute for Microbial Diseases, Osaka University, Suita, Japan; z CREST, Japan Science and Technology Agency, Saitama, Japan; aa International Vaccine Design Center, The Institute of Medical Science, The University of Tokyo, Tokyo, Japan; ab Collaboration Unit for Infection, Joint Research Center for Human Retrovirus infection, Kumamoto University, Kumamoto, Japan; ac MRC-University of Glasgow Centre for Virus Research, Glasgow, UK

**Keywords:** Bat coronavirus, BANAL-20-236, SARS-CoV-2, Spillover

## Abstract

**Background:**

Although several SARS-CoV-2-related coronaviruses (SC2r-CoVs) were discovered in bats and pangolins, the differences in virological characteristics between SARS-CoV-2 and SC2r-CoVs remain poorly understood. Recently, BANAL-20-236 (B236) was isolated from a rectal swab of Malayan horseshoe bat and was found to lack a furin cleavage site (FCS) in the spike (S) protein. The comparison of its virological characteristics with FCS-deleted SARS-CoV-2 (SC2ΔFCS) has not been conducted yet.

**Methods:**

We prepared human induced pluripotent stem cell (iPSC)-derived airway and lung epithelial cells and colon organoids as human organ-relevant models. B236, SARS-CoV-2, and artificially generated SC2ΔFCS were used for viral experiments. To investigate the pathogenicity of B236 *in vivo*, we conducted intranasal infection experiments in hamsters.

**Findings:**

In human iPSC-derived airway epithelial cells, the growth of B236 was significantly lower than that of the SC2ΔFCS. A fusion assay showed that the B236 and SC2ΔFCS S proteins were less fusogenic than the SARS-CoV-2 S protein. The infection experiment in hamsters showed that B236 was less pathogenic than SARS-CoV-2 and even SC2ΔFCS. Interestingly, in human colon organoids, the growth of B236 was significantly greater than that of SARS-CoV-2.

**Interpretation:**

Compared to SARS-CoV-2, we demonstrated that B236 exhibited a tropism toward intestinal cells rather than respiratory cells. Our results are consistent with a previous report showing that B236 is enterotropic in macaques. Altogether, our report strengthens the assumption that SC2r-CoVs in horseshoe bats replicate primarily in the intestinal tissues rather than respiratory tissues.

**Funding:**

This study was supported in part by AMED ASPIRE (JP23jf0126002, to Keita Matsuno, Kazuo Takayama, and Kei Sato); AMED SCARDA Japan Initiative for World-leading Vaccine Research and Development Centers "UTOPIA" (JP223fa627001, to Kei Sato), AMED SCARDA Program on R&D of new generation vaccine including new modality application (JP223fa727002, to Kei Sato); AMED SCARDA Hokkaido University Institute for Vaccine Research and Development (HU-IVReD) (JP223fa627005h0001, to Takasuke Fukuhara, and Keita Matsuno); AMED Research Program on Emerging and Re-emerging Infectious Diseases (JP21fk0108574, to Hesham Nasser; JP21fk0108493, to Takasuke Fukuhara; JP22fk0108617 to Takasuke Fukuhara; JP22fk0108146, to Kei Sato; JP21fk0108494 to G2P-Japan Consortium, Keita Matsuno, Shinya Tanaka, Terumasa Ikeda, Takasuke Fukuhara, and Kei Sato; JP21fk0108425, to Kazuo Takayama and Kei Sato; JP21fk0108432, to Kazuo Takayama, Takasuke Fukuhara and Kei Sato; JP22fk0108534, Terumasa Ikeda, and Kei Sato; JP22fk0108511, to Yuki Yamamoto, Terumasa Ikeda, Keita Matsuno, Shinya Tanaka, Kazuo Takayama, Takasuke Fukuhara, and Kei Sato; JP22fk0108506, to Kazuo Takayama and Kei Sato); AMED Research Program on HIV/AIDS (JP22fk0410055, to Terumasa Ikeda; and JP22fk0410039, to Kei Sato); AMED Japan Program for Infectious Diseases Research and Infrastructure (JP22wm0125008 to Keita Matsuno); AMED CREST (JP21gm1610005, to Kazuo Takayama; JP22gm1610008, to Takasuke Fukuhara; JST PRESTO (JPMJPR22R1, to Jumpei Ito); JST CREST (JPMJCR20H4, to Kei Sato); JSPS KAKENHI Fund for the Promotion of Joint International Research (International Leading Research) (JP23K20041, to G2P-Japan Consortium, Keita Matsuno, Takasuke Fukuhara and Kei Sato); JST SPRING (JPMJSP2108 to Shigeru Fujita); JSPS KAKENHI Grant-in-Aid for Scientific Research C (22K07103, to Terumasa Ikeda); JSPS KAKENHI Grant-in-Aid for Scientific Research B (21H02736, to Takasuke Fukuhara); JSPS KAKENHI Grant-in-Aid for Early-Career Scientists (22K16375, to Hesham Nasser; 20K15767, to Jumpei Ito); JSPS Core-to-Core Program (A. Advanced Research Networks) (JPJSCCA20190008, to Kei Sato); JSPS Research Fellow DC2 (22J11578, to Keiya Uriu); JSPS Research Fellow DC1 (23KJ0710, to Yusuke Kosugi); JSPS Leading Initiative for Excellent Young Researchers (LEADER) (to Terumasa Ikeda); World-leading Innovative and Smart Education (WISE) Program 1801 from the 10.13039/501100001700Ministry of Education, Culture, Sports, Science and Technology (MEXT) (to Naganori Nao); 10.13039/501100003478Ministry of Health, Labour and Welfare (MHLW) under grant 23HA2010 (to Naganori Nao and Keita Matsuno); The Cooperative Research Program (Joint Usage/Research Center program) of Institute for Life and Medical Sciences, Kyoto University (to Kei Sato); International Joint Research Project of the Institute of Medical Science, the 10.13039/501100004721University of Tokyo (to Terumasa Ikeda and Takasuke Fukuhara); The 10.13039/100011313Tokyo Biochemical Research Foundation (to Kei Sato); Takeda Science Foundation (to Terumasa Ikeda and Takasuke Fukuhara); Mochida Memorial Foundation for Medical and Pharmaceutical Research (to Terumasa Ikeda); The Naito Foundation (to Terumasa Ikeda); Hokuto Foundation for Bioscience (to Tomokazu Tamura); 10.13039/100020107Hirose Foundation (to Tomokazu Tamura); and 10.13039/501100004398Mitsubishi Foundation (to Kei Sato).

**Research in context**

**Evidence before this study**

SARS-CoV-2-related coronaviruses (SC2r-CoVs) are carried in wildlife animals such as bats and pangolins, known as the pool of novel zoonotic pathogens. Recently, BANAL-20-236 (B236), a replication-competent SC2r-CoV, was isolated from a rectal swab of Malayan horseshoe bat. In previous studies, only conventional human cell lines and rhesus macaques were used to investigate the characteristics of B236.

**Added value of this study**

Here, we experimentally investigated the growth efficiency of B236 using human respiratory epithelial cells and human colon organoids derived from human induced pluripotent stem cells. Compared to SARS-CoV-2, B236 exhibited a tropism toward intestinal cells rather than respiratory cells. We further demonstrated that B236 was less pathogenic than SARS-CoV-2 in hamsters.

**Implications of all the available evidence**

To prevent future pandemics, it is essential to understand the virological characteristics of SC2r-CoVs such as B236. Advanced culture systems like human organoids allow us to characterize SC2r-CoVs in more biologically realistic environments. This would elucidate the principles of how the virus spills over from wild animals and causes pandemics.

## Introduction

Severe acute respiratory syndrome coronavirus 2 (SARS-CoV-2) emerged at the end of 2019 and caused a pandemic of coronavirus disease 2019 (COVID-19).^1^, ^2^, ^3^ The rapid development of anti-SARS-CoV-2 vaccines and antivirals has greatly contributed to COVID-19 prevention and control. However, the emergence of several SARS-CoV-2 variants of concern (VOCs), such as Alpha, Delta, and Omicron, has complicated the continuous efforts to control the spread of SARS-CoV-2.^4^^,^^5^

The origin of SARS-CoV-2 is an issue in the field of virology. Importantly, the discovery of SARS-CoV-2-related coronaviruses (SC2r-CoVs) in feces or rectal swabs of horseshoe bats (genus *Rhinolophus*) (e.g., RaTG13,^6^ RmYN02,^7^ RacCS203,^8^ RpYN06,^9^ RshSTT182,^10^ Rc-o319,^11^ Rp22DB159^12^) provided evidence supporting that SARS-CoV-2 originated from the zoonotic transmission of certain SC2r-CoV into the human population. A clear difference between SARS-CoV-2 and SC2r-CoVs is the presence of the furin cleavage site (FCS) only in the spike (S) protein of SARS-CoV-2. In other words, SC2r-CoVs harboring the FCS have not been identified yet. Previous papers showed that the FCS has important roles for SARS-CoV-2 pathogenicity and transmissibility by the respiratory route in experimental animal models.^13^^,^^14^

Recently, BANAL-20-236 (B236), a replication-competent SC2r-CoV, was successfully isolated from the rectal swab of Malayan horseshoe bat (*Rhinolophus marshalli*).^15^ This enabled us to experimentally investigate the virological features of a bat SC2r-CoV. Importantly, B236 can utilize human angiotensin converting enzyme 2 (ACE2) as an infection receptor with higher binding affinity than early isolates of SARS-CoV-2.^15^ This observation suggests the existence of SC2r-CoVs in the wild that can be transmitted to humans without additional adaptive evolution. In other words, the SC2r-CoVs in wild bats, which have the potential to infect humans, could cause another outbreak or pandemic in the future. To prepare for future coronavirus outbreaks, it is important to elucidate the virological characteristics of human ACE2-adapted SC2r-CoVs such as B236.

In this study, we investigated the virological characteristics of B236 using *in vitro* human cell cultures and experimental hamsters and compared them with those of SARS-CoV-2.

## Methods

### Ethics statement

All experiments with hamsters were performed in accordance with the Science Council of Japan's Guidelines for the Proper Conduct of Animal Experiments. The protocols were approved by the Institutional Animal Care and Use Committee of National University Corporation Hokkaido University (approval ID: 22-0114).

### Cell culture

HEK293 cells (a human embryonic kidney cell line; ATCC, CRL-1573) and HOS-ACE2/TMPRSS2 cells (human osteosarcoma HOS cells stably expressing human ACE2 and TMPRSS2)^16^^,^^17^ were maintained in DMEM (high glucose) (Sigma–Aldrich, Cat# 6429-500ML) containing 10% fetal bovine serum (FBS, Sigma–Aldrich Cat# 172012-500ML) and 1% penicillin–streptomycin (PS) (Sigma–Aldrich, Cat# P4333-100ML). HEK293-ACE2 cells (HEK293 cells stably expressing human ACE2)^18^ were maintained in DMEM (high glucose) containing 10% FBS, 1 μg/ml puromycin (InvivoGen, Cat# ant-pr-1) and 1% PS. Vero cells [an African green monkey (*Chlorocebus sabaeus*) kidney cell line; JCRB Cell Bank, JCRB0111] were maintained in Eagle's minimum essential medium (EMEM) (Sigma–Aldrich, Cat# M4655-500ML) containing 10% FBS and 1% PS. VeroE6/TMPRSS2 cells (VeroE6 cells stably expressing human TMPRSS2; JCRB Cell Bank, JCRB1819)^19^ were maintained in DMEM (low glucose) (Wako, Cat# 041-29775) containing 10% FBS, G418 (1 mg/ml; Nacalai Tesque, Cat# G8168-10ML) and 1% PS. Calu-3 cells (ATCC, HTB-55) were maintained in Eagle's minimum essential medium (EMEM) (Sigma–Aldrich, Cat# M4655-500ML) containing 10% FBS and 1% PS. Human airway and lung epithelial cells derived from human induced pluripotent stem cells (iPSCs) were manufactured according to established protocols as described below (see “Preparation of human airway and lung epithelial cells from human iPSCs” section) and provided by HiLung Inc. The human airway organoid-derived air-liquid interface (AO-ALI) model was generated according to established protocols as described below (see “AO-ALI model” section). Caco-2 cells (a human colorectal adenocarcinoma cell line; RIKEN BRC, RCB0988) were cultured with EMEM (Sigma–Aldrich, Cat# M4655-500ML) containing 10% FBS and 1% PS, and 1 × GlutaMAX (Thermo Fisher Scientific, #35050061). Human iPSC-derived colon organoids were manufactured according to established protocols as described below (see “Preparation of human iPSC-derived colon organoids” section).

### Preparation of human iPSC-derived colon organoids

The iPSC line, 1383D6, was maintained on 0.5 μg/cm^2^ recombinant human laminin 511 E8 fragments (iMatrix-511; Cat# 892012; Nippi) with StemFit AK02N medium (Cat# RCAK02N; Ajinomoto) containing 10 μM Y-27632 (Cat# 034-24024; FUJIFILM Wako Pure Chemical). To passage the cells, iPSC colonies were treated with TrypLE Select Enzyme (Cat# 12563029; Thermo Fisher Scientific) for 10 min at 37 °C. After centrifugation, cells were seeded on Matrigel® Growth Factor Reduced Basement Membrane (Cat# 354230; Corning)-coated cell culture plates (2.0 × 10^5^ cells/4 cm^2^) and cultured for 2 d. To perform definitive endoderm differentiation, human iPSCs were treated with 100 ng/ml Activin A (Cat# 338-AC-01M; R&D Systems) and 10 μM Y-27632 in RPMI1640 medium (Cat# R8758-500; Sigma–Aldrich) supplemented with 1 × B-27 Supplement Minus Vitamin A (Cat# 12587001; Thermo Fisher Scientific), 1 × GlutaMAX (Cat# 35050-079; Thermo Fisher Scientific), and 1 × PS for 3 d. To perform hindgut differentiation, the cells were treated with 200 ng/ul FGF2 (Cat# 160-0010-3; Katayama Chemical Industries) in RPMI1640 medium supplemented with 1 × B-27 Supplement Minus Vitamin A, 1 × GlutaMAX, and 1 × penicillin-streptomycin for 4 d. To generate colon organoids, the cells were dissociated and embedded in Matrigel Growth Factor Reduced Basement Membrane to generate organoids. To perform colonic differentiation, the cells were treated with 3 μM CHIR99021 (Cat# 034-23103; FUJIFILM Wako Pure Chemical), 0.5 μM A-83-01 (Cat# 035-24113; FUJIFILM Wako Pure Chemical), 50 ng/ml Noggin (Cat# 120-10C; PeproTech), 30 ng/ul Forskolin (Cat# 063-02193; FUJIFILM Wako Pure Chemical), and 50 ng/ml EGF (Cat# AF-100-15; PeproTech) in advanced DMEM/F12 supplemented with 1 × N2 (Cat# 141-08941; FUJIFILM Wako Pure Chemical), 1 × B-27 Supplement Minus Vitamin A, 1 × GlutaMAX, 0.05% bovine serum albumin, and 1 × penicillin-streptomycin for 13 d. To perform the infection experiments, the colon organoids were recovered from Matrigel, and the suspension of colon organoids (small free-floating clumps) was seeded onto Matrigel-coated 24-well cell culture plates (1.0 × 10^5^ cells/2 cm^2^) and cultured for 3 d.

### AO-ALI model

An airway organoid (AO) model was generated according to our previous report.^20^, ^21^, ^22^, ^23^ Briefly, normal human bronchial epithelial cells (NHBEs, Cat# CC-2540, Lonza) were used to generate AOs. NHBEs were suspended in 10 mg/ml cold Matrigel growth factor reduced basement membrane matrix (Corning, Cat# 354230). Fifty microliters of cell suspension were solidified on prewarmed cell culture-treated multiple dishes (24-well plates; Thermo Fisher Scientific, Cat# 142475) at 37 °C for 10 m, and then, 500 μl of expansion medium was added to each well. AOs were cultured with AO expansion medium for 10 d. For maturation of the AOs, expanded Aos were cultured with AO differentiation medium for 5 d.

**Fig. 1:**
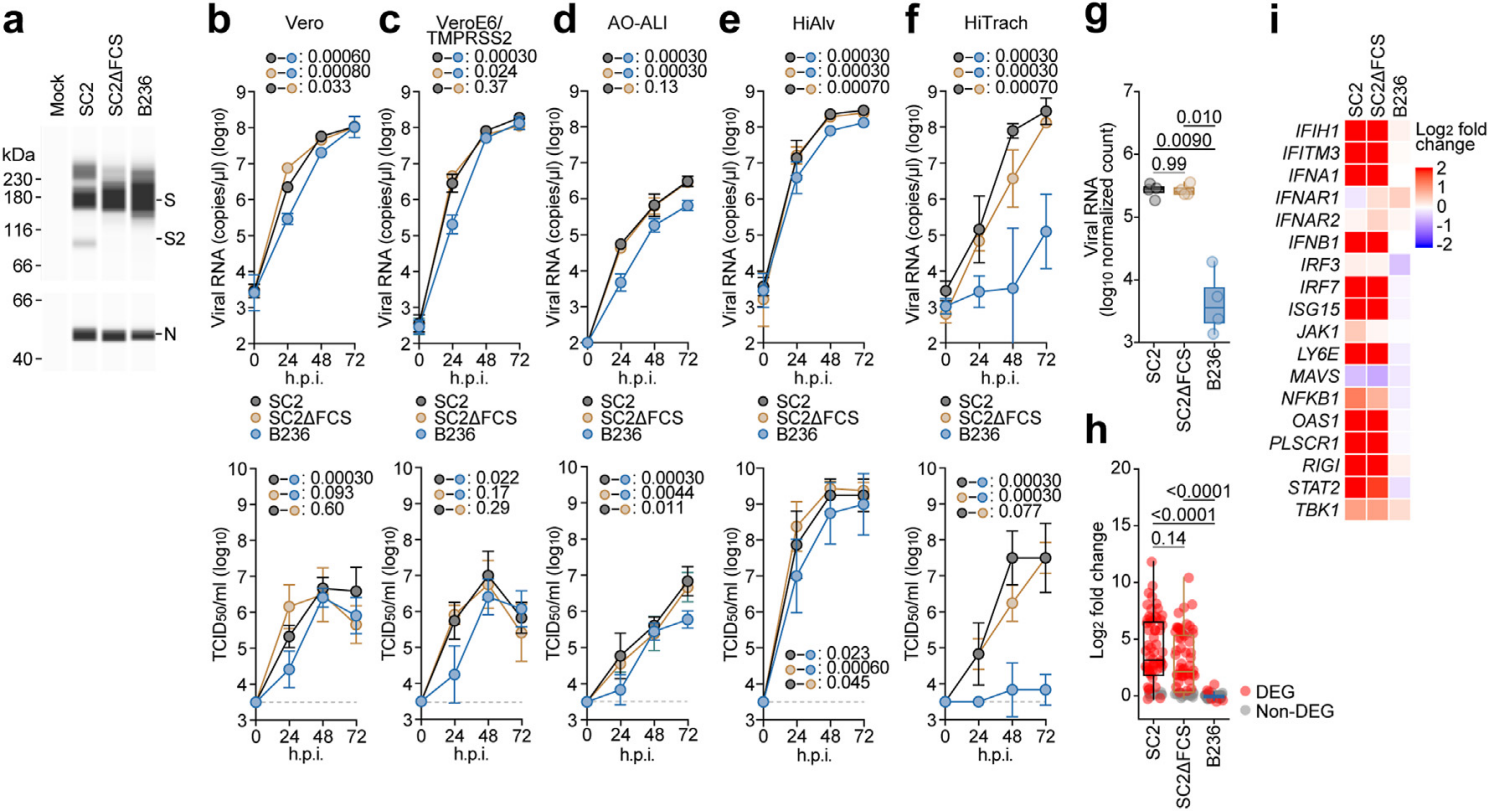
**B236 replicated in respiratory cell cultures less efficiently than SARS-CoV-2**. (**a**) Representative western blots of purified SARS-CoV-2 (SC2), FCS-deleted SARS-CoV-2 (SC2ΔFCS) and BANAL-20-236 (B236) virions from VeroE6/TMPRSS2 cells stained with anti-S2 (top) and anti-nucleocapsid (bottom) antibodies. (**b–f**) Viral growth assay. SC2, SC2ΔFCS and B236 were inoculated into Vero cells (**b**), VeroE6/TMPRSS2 cells (**c**), human airway organoid-derived air-liquid interface (AO-ALI) system (**d**), human induced pluripotent stem cell (iPSC)-derived lung alveolar cells (HiAlv) (**e**) and human induced pluripotent stem cell (iPSC)-derived airway epithelial cells (HiTrach) (**f**). The copy numbers of viral RNA in the culture supernatant (**b and c**) and the apical sides of cultures (**d-f**) were routinely quantified by RT-qPCR (top). The viral titer [50% tissue culture infectious dose (TCID_50_)] was also measured by the culture supernatant (**b and c**) and the apical sides of cultures (**d-f**) (bottom). The horizontal dashed line indicates the detection limit (10^3.5^ TCID_50_/ml). Assays were performed in triplicate (**d**) or quadruplicate (**b, c, e and f**). The presented data are expressed as the average ± 95% confidence intervals (CI). In (**b–f)**, statistically significant differences across timepoints were determined by multiple regression. The familywise error rates (FWERs) calculated using the Holm method (**b–f**) are indicated in the figures. (**g**) The log normalized count of SARS-CoV-2 reads in the SC2- and SC2ΔFCS-infected HiTrach iPSC-derived airway epithelial cells and that of B236 reads in the B236-infected cells. The count is normalized to a million unit and then log transformed. n = 4. Numbers above the box plots are adjusted *P* values calculated by Games–Howell test. (**h**) The log_2_ expression fold change of genes listed in the GO Biological Process term “cellular response to type I interferon” (GO:0071357) in the HiTrach iPSC-derived airway epithelial cells infected with SC2, SC2ΔFCS, and B236. Each circle indicates a data point. Red circle, DEG; grey circle, non-DEG. Numbers above the box plots are adjusted *P* values calculated by Dunn test. (**i**) The log_2_ expression fold change of genes related to the suppression of SARS-CoV-2 replication^24^, ^25^, ^26^, ^27^, ^28^ in the HiTrach iPSC-derived airway epithelial cells infected with SC2, SC2ΔFCS, and B236.

The AO-ALI model (Fig. 1d) was generated according to our previous.^21^^,^^29^^,^^30^ For generation of AO-ALI, expanding AOs were dissociated into single cells, and then were seeded into Transwell inserts (Corning, Cat# 3413) in a 24-well plate. AO-ALI was cultured with AO differentiation medium for 5 d to promote their maturation. AO-ALI was infected with SARS-CoV-2 from the apical side.

### Preparation of human airway and lung epithelial cells from human iPSCs

The air-liquid interface culture of the human iPSC-derived lung alveolar cells (HiAlv) and iPSC-derived airway epithelial cells (HiTrach) (Fig. 1e and f) was differentiated from human iPSC-derived lung progenitor cells as previously described.^21^^,^^23^^,^^30^, ^31^, ^32^, ^33^, ^34^ Briefly, lung progenitor cells were induced stepwise from human iPSCs according to a 21-d and 4-step protocol.^32^ At day 21, lung progenitor cells were isolated with the specific surface antigen carboxypeptidase M and seeded onto the upper chamber of a 24-well Cell Culture Insert (Falcon, #353104), by 28-day and 7-day differentiation of airway and lung epithelial cells, respectively. PneumaCult ALI (STEMCELL Technologies, Cat# ST-05001) with heparin (Nacalai Tesque, Cat# 17513-96) and Y-27632 (LC Laboratories, Cat# Y-5301) hydrocortisone (Sigma–Aldrich, Cat# H0135) was used for induction of airway epithelial cells. Alveolar differentiation medium with dexamethasone (Sigma–Aldrich, Cat# D4902), KGF (PeproTech, Cat# 100-19), 8-Br-cAMP (BIOLOG Life Science Institute, Cat# B007), 3-isobutyl 1-methylxanthine (IBMX) (Fujifilm Wako, Cat# 095-03413), CHIR99021 (Axon Medchem, Cat# 1386), and SB431542 (Fujifilm Wako, Cat# 198-16543) was used for the induction of lung epithelial cells.

### Plasmid construction

Plasmids expressing the codon-optimized SARS-CoV-2 (SC2) S proteins (strain Wuhan-Hu-1; GenBank accession number: NC_045512.2)^2^ was kindly provided from Prof. Kenzo Tokunaga. Plasmids expressing the codon-optimized S proteins of BANAL-20-236 (B236) (GISAID ID: EPI_ISL_4302647 and GenBank accession: MZ937003.2) were prepared in our previous study.^35^ Plasmids expressing the codon-optimized FCS-deleted SC2ΔFCS were generated by site-directed overlap extension PCR using the following primers: pC-S PRRA681-4del Fw, 5′-CCC AGA CCA ACA GCA GGT CTG TGG CAA GCC-3’; pC-S PRRA681-4del Rv, 5′-GGC TTG CCA CAG ACC TGC TGT TGG TCT GGG-3’; pCS-SC2-Fw, 5′-CAC TAT AGG GCG AAT TGG GTA CCA TGT TTG TGT TCC TGG T-3’; pCS-SC2-Rv, 5′-AGC TCC ACC GCG GTG GCG GCC GCT CAG GTG TAG TGC AGT TTC A-3’. The resulting PCR fragment was digested with KpnI (New England Biolabs, Cat# R0142S) and NotI (New England Biolabs, Cat# R1089S) and inserted into the corresponding site of the pCAGGS vector.^36^ Nucleotide sequences were determined by DNA sequencing services (Eurofins), and the sequence data were analyzed by Sequencher v5.1 software (Gene Codes Corporation).

### Western blot analysis

Western blotting was performed as previously described with some modifications.^37^ VeroE6/TMPRSS2 cells infected with SARS-CoV-2, SC2ΔFCS, B236 (m.o.i. = 0.01) and the cell culture supernatants were corrected at 48 h.p.i. The supernatants were layered onto 500 μl 20% sucrose in PBS and centrifuged at 20,000 g for 2 h at 4 °C. Pelleted virions were resuspended in 1 × sample buffer [50 mM Tris–HCl (pH 6.8), 2% SDS, 6% β-mercaptoethanol, 10% glycerol, 0.0025% bromophenol blue] and boiled for 10 m. After cooling down, viral lysates were mixed with diluted sample buffer (proteinsimple, Cat# 99351). Then, 5 × Fluorescent Master mix (proteinsimple, Cat# PS-ST01EZ-8) was added at a ratio of 4:1. Simple Western System was used for protein analysis. For protein detection, the following antibodies were used: mouse anti-SARS-CoV-2 S2 monoclonal antibody (R&D Systems, Cat# MAB10557, 1:50), rabbit anti-SARS-CoV-2 N polyclonal antibody (GeneTex, GTX135570, 1:1000), anti-mouse secondary antibody (proteinsimple, Cat# 042-205), and anti-rabbit secondary antibody (proteinsimple, Cat# 042-206). The amount of virus lysate applied was normalized to that of viral N protein. Bands were visualized and analyzed using Compass for Simple Western v6.1.0 (proteinsimple).

### SARS-CoV-2 reverse genetics

Recombinant FCS-deleted SARS-CoV-2 (SC2ΔFCS) was generated by circular polymerase extension reaction (CPER) as previously described.^18^^,^^38^^,^^39^ In brief, 9 DNA fragments encoding the partial genome of SARS-CoV-2 (strain WK-521, PANGO lineage A; GISAID ID: EPI_ISL_408667)^19^ were prepared by PCR using PrimeSTAR GXL DNA polymerase (Takara, Cat# R050A). A linker fragment encoding hepatitis delta virus ribozyme, bovine growth hormone poly A signal and cytomegalovirus promoter was also prepared by PCR. The 10 obtained DNA fragments were mixed and used for CPER.^39^ To prepare FCS-deleted replication-competent recombinant SARS-CoV-2, we used fragment 8, in which the FCS (PRRA681-684del) gene was deleted in the Spike gene. The template plasmid for mutated fragment 8 was generated by site-directed overlap extension PCR using the following primers: PRRA681-4del Fw, 5′-CAG ACT CAG ACT AAT TCT CGT AGT GTA GCT AGT CAA TCC-3’; PRRA681-4del Rv, 5′-GGA TTG ACT AGC TAC ACT ACG AGA ATT AGT CTG AGT CTG-3’; F8-Fw, 5′-ACA CGC TAC CGG TCT CGA GAC CAT GAT GTT TGT TTT TCT T-3’; F8-Rv, 5′-CAT TGG TCT TAA AGG TAC CTG AGG TGT GAC TGG AAA ACC C-3’. Nucleotide sequences were determined by a DNA sequencing service (Fasmac), and the sequence data were analyzed by Sequencher software v5.1 (Gene Codes Corporation).

To produce recombinant SARS-CoV-2 (seed viruses), the CPER products were transfected into HEK293-C34 cells using TransIT-LT1 (Takara, Cat# MIR2300) according to the manufacturer's protocol. At one day posttransfection, the culture medium was replaced with DMEM (high glucose) (Sigma–Aldrich, Cat# 6429-500 ML) containing 2% FBS, 1% PS and doxycycline (1 μg/ml; Takara, Cat# 1311N). At 6 d posttransfection, the culture medium was harvested and centrifuged, and the supernatants were collected as the seed virus. To remove the CPER products (i.e., SARS-CoV-2-related DNA), 1 ml of the seed virus was treated with 2 μl TURBO Dnase (Thermo Fisher Scientific, Cat# AM2238) and incubated at 37 °C for 1 h. Complete removal of the CPER products from the seed virus was verified by PCR. The working virus stock was prepared using the seed virus as described below (see “SARS-CoV-2 and BANAL-20-236 preparation and titration” section).

### SARS-CoV-2 and B236 preparation and titration

The working virus stocks of SC2, SC2ΔFCS and B236 were prepared and titrated as previously described.^18^^,^^20^^,^^21^^,^^23^^,^^30^^,^^31^^,^^37^^,^^38^^,^^40^, ^41^, ^42^ In this study, clinical isolates of a parental SARS-CoV-2 WK-521 strain^19^ (GISAID ID: EPI_ISL_408667) and bat isolated BANAL-20-236^15^ (GISAID ID: EPI_ISL_4302647 and GenBank accession: MZ937004), recombinant SC2ΔFCS (see “SARS-CoV-2 reverse genetics” section), Delta (B.1.617.2, strain TKYTK1734; GISAID ID: EPI_ISL_2378732)^38^ and Omicron BA.1 lineage (strain TY38-873; GISAID ID: EPI_ISL_7418017)^43^ were used. In brief, 20 μl of the seed virus was inoculated into VeroE6/TMPRSS2 cells (5,000,000 cells in a T-75 flask). One h.p.i., the culture medium was replaced with DMEM (low glucose) (Wako, Cat# 041-29775) containing 2% FBS and 1% PS. At 3 days postinfection (d.p.i.), the culture medium was harvested and centrifuged, and the supernatants were collected as the working virus stock.

The titer of the prepared working virus was measured as the 50% tissue culture infectious dose (TCID_50_). Briefly, one day before infection, VeroE6/TMPRSS2 cells (10,000 cells) were seeded into a 96-well plate. Serially diluted virus stocks were inoculated into the cells and incubated at 37 °C for 4 d. The cells were observed under a microscope to judge the CPE appearance. The value of TCID_50_/ml was calculated with the Reed–Muench method.^44^

For verification of the sequences of SC2, SC2ΔFCS and B236 working viruses, viral RNA was extracted from the working viruses using a QIAamp viral RNA mini kit (Qiagen, Cat# 52906) and viral genome sequences were analyzed as described below (see “Viral genome sequencing” section).

### Viral genome sequencing

Viral genome sequencing was performed as previously described.^31^ Briefly, the virus sequences were verified by viral RNA-sequencing analysis. Viral RNA was extracted using a QIAamp viral RNA mini kit (Qiagen, Cat# 52906). The sequencing library employed for total RNA sequencing was prepared using the NEB Next Ultra RNA Library Prep Kit for Illumina (New England Biolabs, Cat# E7530). Paired-end 76-bp sequencing was performed using a MiSeq system (Illumina) with MiSeq reagent kit v3 (Illumina, Cat# MS-102-3001). Sequencing reads were trimmed using fastp v0.21.0^45^ and subsequently mapped to the viral genome sequences of a lineage A isolate (strain WK-521, GISAID ID: EPI_ISL_408667)^19^ or BANAL-20-236 (GISAID ID: EPI_ISL_4302647 and GenBank accession: MZ937004)^15^ using BWA-MEM v0.7.17.^46^ Variant calling, filtering, and annotation were performed using SAMtools v1.9^47^ and snpEff v5.0e.^48^ Information on the unexpected substitutions detected is summarized in Supplementary Table S7).

### Airway-on-a-chip

**Fig. 2:**
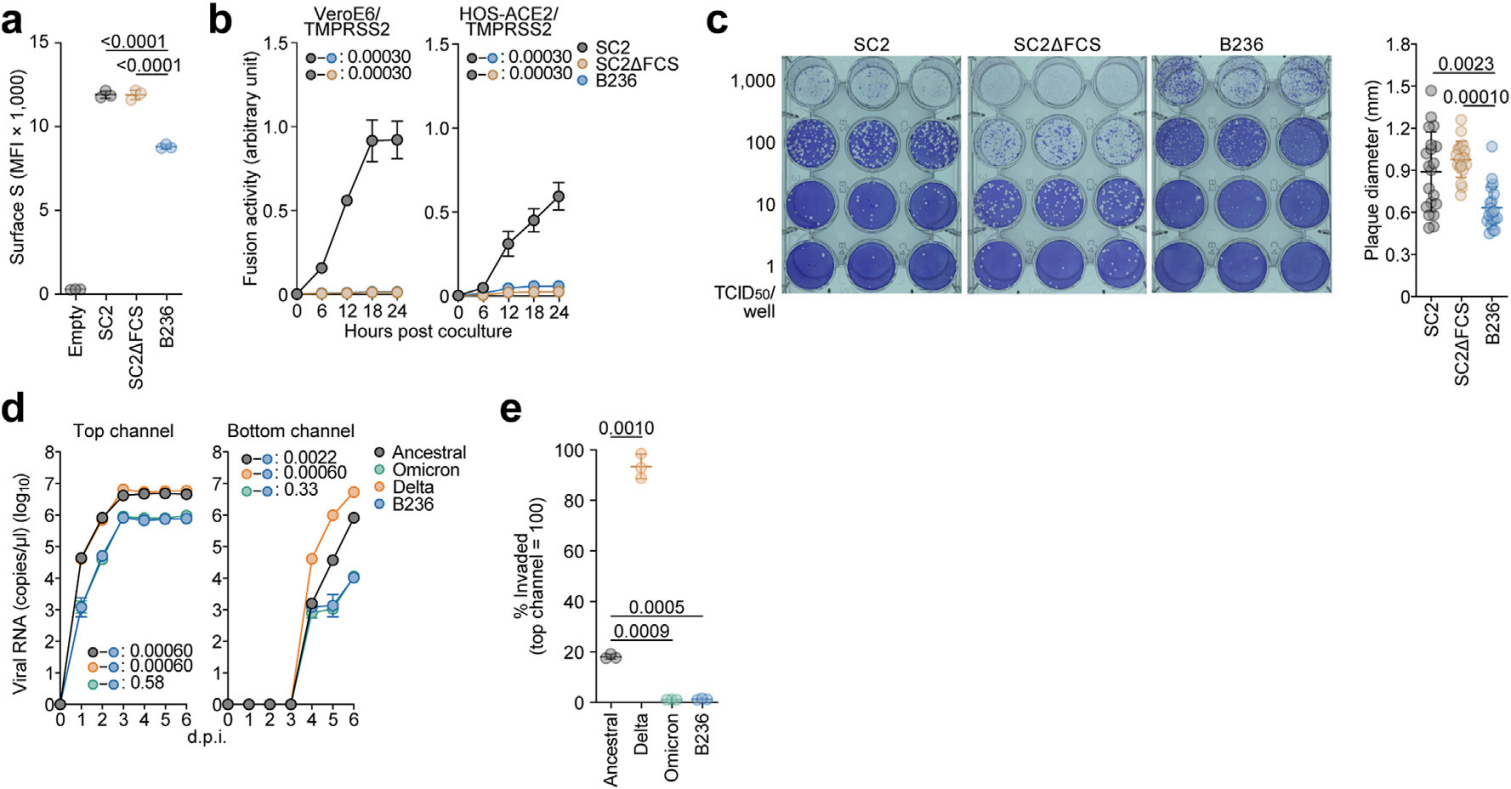
**B236 is less fusogenic than SARS-CoV-2**. (**a–b**) S-based fusion assay. S protein expression on the cell surface (**a**). The summarized data are shown. S-based fusion assay in VeroE6/TMPRSS2 cells and HOS-ACE2/TMPRSS2 cells (**b**). The recorded fusion activity (arbitrary units; *Renilla* luciferase activity per the surface S MFI) is shown. (**c**) A plaque assay was performed using VeroE6/TMPRSS2 cells. Left, representative figures. Right, the summary of the size of plaques (n = 20 for each virus). Each dot indicates the diameter of the respective plaque. (**d, e**) Viral growth in an airway-on-a-chip system. An ancestral SARS-CoV-2 WK-521 strain (Wuhan), Omicron BA.1 (Omicron), Delta variants, and BANAL-20-236 (B236) were inoculated into an airway-on-a-chip system. The copy numbers of viral RNA in the top (**d, left**) and bottom (**d, right**) channels of an airway-on-a-chip were routinely quantified by RT-qPCR. In (**e)**, the percentage of viral RNA load in the bottom channel per top channel at 6 d.p.i. (i.e., % invaded virus from the top channel to the bottom channel) is shown. Assays were performed in triplicate (**a, d, e**) or quadruplicate (**b**). The presented data are expressed as the average ± standard deviation (SD). In (**a**, **e)**, each dot indicates the result of an individual replicate. Statistically significant differences versus SC2 or SC2ΔFCS were determined by a two-sided Student's t test (**a**) or a two-sided Mann–Whitney U test (**c**). In (**b)**, statistically significant differences across timepoints were determined by multiple regression. The familywise error rates (FWERs) calculated using the Holm method are indicated in the figures. In (**e**), statistically significant differences were determined by a two-sided Welch's test.

Airway-on-a-chip (Fig. 2d and e) was prepared as previously described.^20^, ^21^, ^22^, ^23^^,^^30^ Human lung microvascular endothelial cells (HMVEC-L) were obtained from Lonza (Cat# CC-2527) and cultured with EGM-2-MV medium (Lonza, Cat# CC-3202). For preparation of the airway-on-a-chip, first, the bottom channel of a polydimethylsiloxane (PDMS) device was precoated with fibronectin (3 μg/ml, Sigma–Aldrich, Cat# F1141). The microfluidic device was generated according to our previous report.^49^ HMVEC-L cells were suspended at 5,000,000 cells/ml in EGM2-MV medium. Then, 10 μl of suspension medium was injected into the fibronectin-coated bottom channel of the PDMS device. Then, the PDMS device was turned upside down and incubated. After 1 h, the device was turned over, and the EGM2-MV medium was added into the bottom channel. After 4 d, AOs were dissociated and seeded into the top channel. AOs were generated according to our previous report.^29^ AOs were dissociated into single cells and then suspended at 5,000,000 cells/ml in the AO differentiation medium. Ten microliter suspension medium was injected into the top channel. After 1 h, the AO differentiation medium was added to the top channel. In the infection experiments (Fig. 2d and e), the AO differentiation medium containing either SARS-CoV-2 (WK-521, Omicron BA.1 and Delta variants) and B236 (500 TCID_50_) was inoculated into the top channel. At 2 h.p.i., the top and bottom channels were washed and cultured with AO differentiation and EGM2-MV medium, respectively. The culture supernatants were collected, and viral RNA was quantified using RT-qPCR (see “RT-qPCR” section above).

### Microfluidic device

A microfluidic device was generated according to our previous report.^21^^,^^49^ Briefly, the microfluidic device consisted of two layers of microchannels separated by a semipermeable membrane. The microchannel layers were fabricated from PDMS using a soft lithographic method. PDMS prepolymer (Dow Corning, Cat# SYLGARD 184) at a base to curing agent ratio of 10:1 was cast against a mold composed of SU-8 2150 (MicroChem, Cat# SU-8 2150) patterns formed on a silicon wafer. The cross-sectional size of the microchannels was 1 mm in width and 330 μm in height. Access holes were punched through the PDMS using a 6-mm biopsy punch (Kai Corporation, Cat# BP-L60K) to introduce solutions into the microchannels. Two PDMS layers were bonded to a PET membrane containing 3.0-μm pores (Falcon, Cat# 353091) using a thin layer of liquid PDMS prepolymer as the mortar. PDMS prepolymer was spin-coated (4000 rpm for 60 s) onto a glass slide. Subsequently, both the top and bottom channel layers were placed on the glass slide to transfer the thin layer of PDMS prepolymer onto the embossed PDMS surfaces. The membrane was then placed onto the bottom layer and sandwiched with the top layer. The combined layers were left at room temperature for one day to remove air bubbles and then placed in an oven at 60 °C overnight to cure the PDMS glue. The PDMS devices were sterilized by placing them under UV light for 1 h before the cell culture.

### Virus infection

One day before infection, Vero cells (10,000 cells), VeroE6/TMPRSS2 cells (10,000 cells) and Caco-2 cells (20000 cells) were seeded into a 96-well plate. SC2, SC2ΔFCS or B236 [1000 TCID_50_ for Vero cells (Fig. 1b); 100 TCID_50_ for VeroE6/TMPRSS2 cells (Fig. 1c); 2000 TCID_50_ for Caco-2 cells (Fig. 4a)] and was inoculated and incubated at 37 °C for 1 h. The infected cells were washed, and 180 μl of culture medium was added. The culture supernatant (10 μl) was harvested at the indicated timepoints and used for RT-qPCR to quantify the viral RNA copy number (see “RT-qPCR” section below). In the infection experiments using AO-ALI (Fig. 1d), human iPSC-derived airway (HiTrach) and lung (HiAlv) cells (Fig. 1e and f), working viruses were diluted with Opt-MEM (Thermo Fisher Scientific, Cat# 11058021). The diluted viruses (1000 TCID_50_ in 100 μl) were inoculated onto the apical side of the culture and incubated at 37 °C for 1 h. The inoculated viruses were removed and washed twice with Opti-MEM. For collection of the viruses, 100 μl Opti-MEM was applied onto the apical side of the culture and incubated at 37 °C for 10 min. The Opti-MEM was collected and used for RT-qPCR to quantify the viral RNA copy number (see “RT-qPCR” section below).

**Fig. 3:**
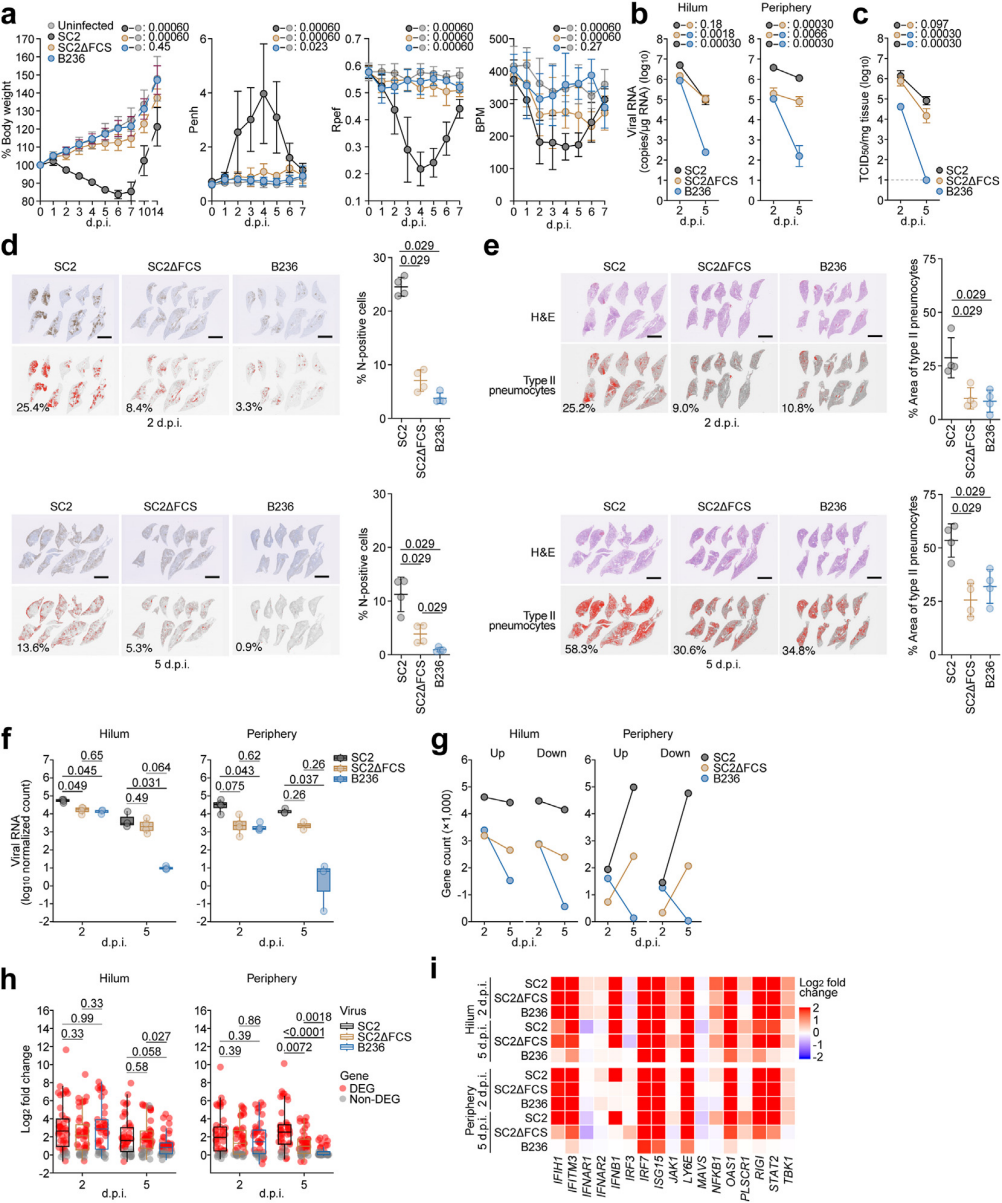
**B236 is less pathogenic than SARS-CoV-2**. Viral growth and pathogenicity in hamster models. Syrian hamsters were intranasally inoculated with SARS-CoV-2 (SC2), FCS-deleted SARS-CoV-2 (SC2ΔFCS) and BANAL-20-236 (B236). Six hamsters of the same age were intranasally inoculated with saline (uninfected). Six hamsters per group were used to routinely measure the respective parameters (**a**). Four hamsters per group were euthanized at 2 and 5 d.p.i. and used for virological and pathological analysis (**b–e**). (**a**) Body weight, enhanced pause (Penh), the ratio of time to peak expiratory flow relative to the total expiratory time (Rpef) and frequency of breath (BPM) values of infected hamsters (n = 6 per infection group). (**b**) Viral RNA loads in the lung hilum (left) and lung periphery (right) of infected hamsters (n = 4 per infection group). (**c**) Viral titer [50% tissue culture infectious dose (TCID_50_)] in the lung periphery of infected hamsters (n = 4 per infection group). The horizontal dashed line indicates the detection limit (10^1^ TCID_50_/mg tissue). (**d**) Immunohistochemical (IHC) analysis of the SARS-CoV-2 or BANAL-20-236 N protein in the lungs of infected hamsters at 2 d.p.i. (top) and 5 d.p.i (bottom) (4 hamsters per infection group). In each panel, representative figures of IHC staining (top) and the digitalized N-positive area (bottom, indicated in red) are shown. The red numbers in the bottom panels indicate the percentage of the N-positive area. The percentage of N-positive cells in whole lung lobes (right, n = 4 per infection group) are shown. (**e**) Type II pneumocytes in the lungs of infected hamsters at 2 d.p.i. (top) and 5 d.p.i (bottom) (4 hamsters per infection group). In each panel, representative figures of H&E staining (top) and the digitalized inflammatory area with type II pneumocytes (bottom, indicated in red) are shown. The red numbers in the bottom panels indicate the percentage of inflammatory area with type II pneumocytes. The percentage of type II pneumocytes in whole lung lobes (right, n = 4 per infection group) are shown. In (**a–c)**, data are presented as the average ± 95% CI. In (**a–c)**, statistically significant differences across timepoints were determined by multiple regression. In (**a)**, the 0 d.p.i. data were excluded from the analyses. The familywise error rates (FWERs) calculated using the Holm method are indicated in the figures. In (**d, e**), each dot indicates the result of an individual hamster. In (**d, e**), the statistically significant differences between SC2 and SC2ΔFCS, B236 were determined by a two-sided Mann–Whitney U test. Scale bars, 5 mm (**d, e**). (**f**) The log normalized count of SARS-CoV-2 reads in the SC2- and SC2ΔFCS-infected hamster lung hila and peripheries at 2 and 5 d.p.i. and that of B236 reads in the B236-infected lung hilum and periphery at 2 and 5 d.p.i. The count is normalized to a million unit and then log transformed. n = 4, except for certain groups where some data were excluded due to RNA quality (see Supplementary Table S8). Numbers above the box plots are adjusted *P* values calculated by Dunn test. (**g**) Number of upregulated and downregulated DEGs in the hamster lung hila and peripheries infected with SC2, SC2ΔFCS, and B236 at 2 and 5 d.p.i. (**h**) The log_2_ expression fold change of genes listed in the GO Biological Process term “cellular response to type I interferon” (GO:0071357) in the hamster lung hila and peripheries infected with SC2, SC2ΔFCS, and B236 at 2 and 5 d.p.i. Each circle indicates a data point. Red circle, DEG; grey circle, non-DEG. Numbers above the box plots are adjusted *P* values calculated by Dunn test. (**i**) The log_2_ expression fold change of genes related to the suppression of SARS-CoV-2 replication^24^, ^25^, ^26^, ^27^, ^28^ in the hamster lung hila and peripheries infected with SC2, SC2ΔFCS, and B236 at 2 and 5 d.p.i.

**Fig. 4:**
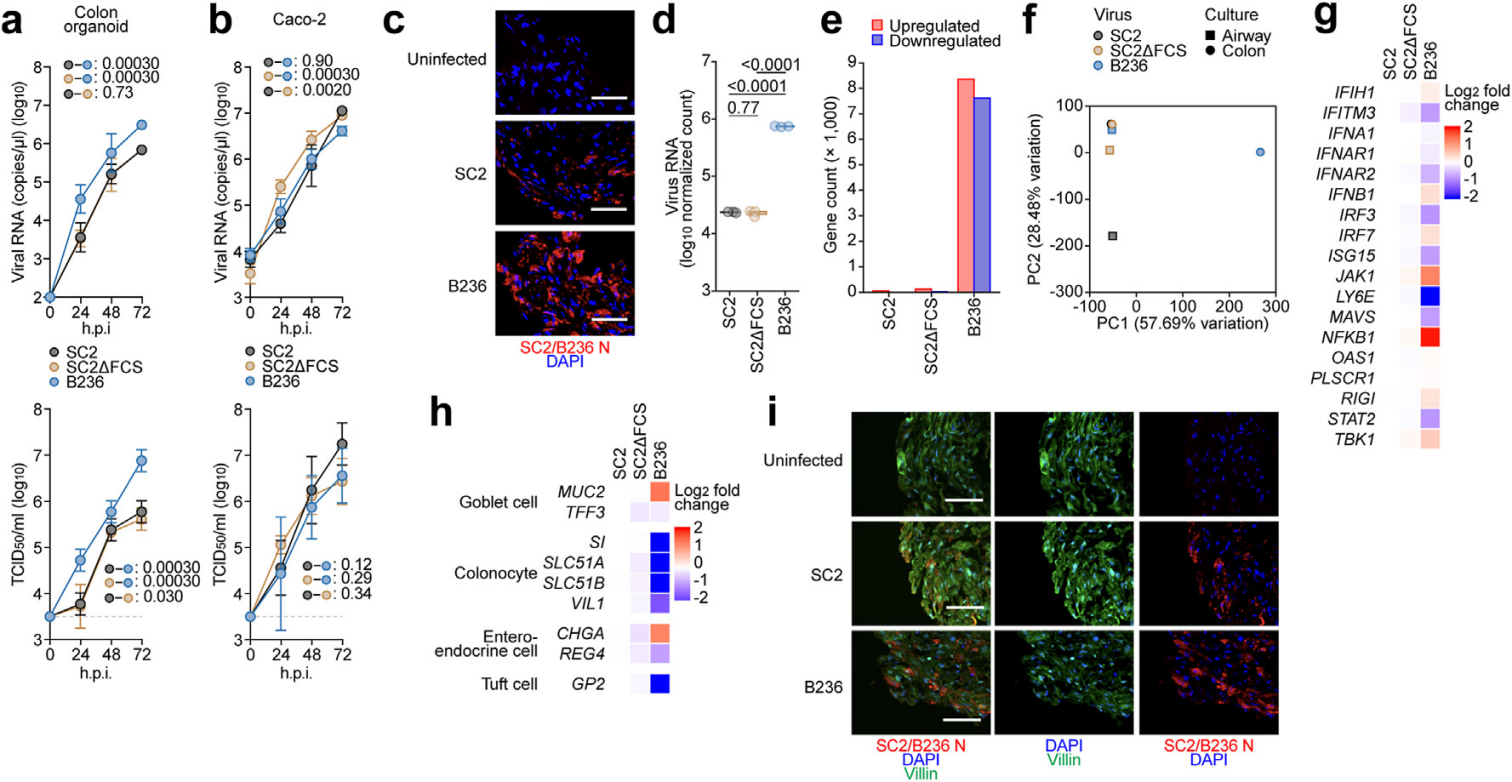
**B236 is more enterotropic than SARS-CoV-2**. (**a-b**) Viral growth assay. SARS-CoV-2 (SC2), FCS-deleted SARS-CoV-2 (SC2ΔFCS), and BANAL-20-236 (B236) were inoculated into human iPSC-derived colon organoids (**a**) and Caco-2 cells (**b**). The copy numbers of viral RNA in the culture supernatants were routinely quantified by RT-qPCR (top). The viral titer [50% tissue culture infectious dose (TCID_50_)] was also measured by the culture supernatant (right). The horizontal dashed line indicates the detection limit (10^3.5^ TCID_50_/ml). Assays were performed in triplicate (**a**) or quadruplicate (**b**). The presented data are expressed as the average ± 95% CI. Statistically significant differences across time points were determined by multiple regression. The familywise error rates (FWERs) calculated using the Holm method are indicated in the figures. (**c**) Immunofluorescence analysis of human iPSC-derived colon organoids stained with SC2/B236 N protein (red). Nuclei were counterstained with DAPI (blue). (**d**) The log normalized count of B236 reads in the B236-infected iPSC-derived colon organoids and that of SARS-CoV-2 reads in the SC2- and SC2ΔFCS-infected colon organoids. The count is normalized to a million unit and then log transformed. n = 3. Numbers above the box plots are adjusted *P* values calculated by Tukey's HSD test. (**e**) Number of upregulated and downregulated DEGs in the colon organoids infected with B236, SC2, and SC2ΔFCS differentially expressed from those in mock-infected colon organoids. (**f**) Principal component (PC) plot of log_2_ expression fold change profile of genes in the HiTrach iPSC-derived airway epithelial cells and colon organoids infected with B236, SC2, and SC2ΔFCS. The number in the PC axis title shows the percentage of variance explained by the PC. The total number of PCs is six. (**g**) The log_2_ expression fold change of genes related to the suppression of SARS-CoV-2 replication in the colon organoids infected with SC2, SC2ΔFCS, and B236. (**h**) The log_2_ expression fold change of marker genes for colonic cells including goblet cell, colonocyte, enteroendocrine cell, and tuft cell. (**i**) Immunofluorescence analysis of human iPSC-derived colon organoids stained with SC2/B236 N protein (red) and villin (green). Nuclei were counterstained with DAPI (blue).

In the infection experiments using human iPSC-derived colon organoids (Fig. 4a), working viruses were diluted with the medium for colon organoids. The diluted viruses (5000 TCID_50_ in 1000 μl) were inoculated and incubated at 37 °C for 1 h. The inoculated viruses were removed and washed once with the medium for colon organoids. The medium was collected and used for RT-qPCR to quantify the viral RNA copy number (see “RT-qPCR” section below). The infection experiments using an airway-on-a-chip system (Fig. 2d and e) were performed as described above (see “Airway-on-a-chip” section).

### RT-qPCR

RT-qPCR was performed as previously described.^18^^,^^20^^,^^21^^,^^23^^,^^30^^,^^31^^,^^37^^,^^38^^,^^40^, ^41^, ^42^ Briefly, 5 μl culture supernatant was mixed with 5 μl of 2 × RNA lysis buffer [2% Triton X-100 (Nacalai Tesque, Cat# 35501-15), 50 mM KCl, 100 mM Tris–HCl (pH 7.4), 40% glycerol, 0.8 U/μl recombinant Rnase inhibitor (Takara, Cat# 2313B)] and incubated at room temperature for 10 min. Rnase-free water (90 μl) was added, and the diluted sample (2.5 μl) was used as the template for real-time RT-PCR performed according to the manufacturer's protocol using One Step TB Green PrimeScript PLUS RT-PCR kit (Takara, Cat# RR096A) and the following primers,which were commonly used for SC2 and B236: Forward *N*, 5′-GCG CAT TGG CAT GGA AG A C-3’; and Reverse *N*, 5′-CTC TGT TGG TGG GAA TGT TTT GT-3’. The viral RNA copy number was standardized with a SARS-CoV-2 direct detection RT-qPCR kit (Takara, Cat# RC300A). Fluorescent signals were acquired using a QuantStudio 1 Real-Time PCR system (Thermo Fisher Scientific), QuantStudio 3 Real-Time PCR system (Thermo Fisher Scientific), QuantStudio 5 Real-Time PCR system (Thermo Fisher Scientific), StepOnePlus Real-Time PCR system (Thermo Fisher Scientific), CFX Connect Real-Time PCR Detection system (Bio-Rad), Eco Real-Time PCR System (Illumina), qTOWER3 G Real-Time System (Analytik Jena), Thermal Cycler Dice Real Time System III (Takara) or 7500 Real-Time PCR System (Thermo Fisher Scientific).

### Host cell gene expression analysis using RT-qPCR

Total RNA was isolated from the colon organoids using ISOGENE (Cat# 319-90211, NIPPON GENE). Human adult and fetal small intestine total RNA, which was used as control, was purchased from BioChain (Cat# R1234226-P, Cat# R1244101, respectively). cDNA was synthesized using 500 ng of total RNA with a Superscript VILO cDNA Synthesis Kit (Cat# 11754250, Thermo Fisher Scientific). Real-time RT-PCR was performed with SYBR Green PCR Master Mix (Cat# 4385614, Thermo Fisher Scientific) using a QuantStudio 3 real-time PCR system (Thermo Fisher Scientific). Relative quantification of target mRNA levels was performed using the 2^−ΔΔCT^ method. Values were normalized to the housekeeping gene *glyceraldehyde 3-phosphate dehydrogenase* (*GAPDH*). The PCR primer sequences are following.

**Table:** 

*ABCC2*	Forward, 5′-TGAGCAAGTTTGAAACGCACAT-3′
	Reverse, 5′-AGCTCTTCTCCTGCCGTCTCT-3′
*ACE2*	Forward, 5′-ACAGTCCACACTTGCCCAAAT-3′
	Reverse, 5′-TGAGAGCACTGAAGACCCATT-3′
*CA2*	Forward, 5′-GGGTACGGCAAACACAACG-3′
	Reverse, 5′-GGCTGTATGAGTGTCGATGTC-3′
*CDX2*	Forward, 5′-TCCGTGTACACCACTCGATATT-3′
	Reverse, 5′-GGAACCTGTGCGAGTGGAT-3′
*CYP2C19*	Forward, 5′-GTCTCTGTCCCAGCTCCAAG-3′
	Reverse, 5′-TTGCTTCCTGATCAAAATGG-3′
*SLC51A*	Forward, 5′-CTGGGCTCCATTGCCATCTT-3′
	Reverse, 5′-CACGGCATAAAACGAGGTGAT-3′
*SLC51B*	Forward, 5′-TCCAGGCAAGCAGAAAAGAAA-3′
	Reverse, 5′-ACTGACAGCACATCTCTCTCT-3′
*TMPRSS2*	Forward, 5′-GTCCCCACTGTCTACGAGGT-3′
	Reverse, 5′-CAGACGACGGGGTTGGAAG-3′
*VIL1*	Forward, 5′-CTGAGCGCCCAAGTCAAAG-3′
	Reverse, 5′-AGCAGTCACCATCGAAGAAGC-3′

### Viral titration of infection experiments

The viral titer of the infection experiments (Fig. 1, Fig. 4b) was measured as the 50% tissue culture infectious dose (TCID_50_). Briefly, one day before infection, VeroE6/TMPRSS2 cells (10,000 cells) were seeded into a 96-well plate. Serially diluted each sample (the culture supernatant or the apical sides of cultures) were inoculated into three (Fig. 1b, c, and 1f), four (Fig. 1, Fig. 4b) or six (Fig. 1, Fig. 4a) lines of the cells and incubated at 37 °C for 4 d. The cells were observed under a microscope to judge the CPE appearance. The value of TCID_50_/ml was calculated with the Reed–Muench method.^44^

### RNA preparation and sequencing

Total RNA was extracted from the human iPSC-derived airway epithelial cells, hamster lung hila and peripheries, and human iPSC-derived colon organoids using QIAamp RNA Blood Mini Kit (Qiagen, Cat# 52304) following the manufacturer's protocols. cDNA library was subsequently synthesized using TruSeq Stranded mRNA Library Prep Kit (Illumina) following the manufacturer's protocols. The library was then sequenced using the Illumina Novaseq6000 sequencing platform. Nucleotide sequences in all samples have a mean quality score ≥34.27, ensuring the overall accuracy of the base calling >99.96% (Supplementary Table S8). Samples with RNA integrity number less that 7 were discarded from downstream analyses (Supplementary Table S8).

### Calculating identity between B236 and SARS-CoV-2

The amino acid sequences of B236 (GenBank accession ID: MZ937003.2) and SARS-CoV-2 (Refseq accession ID: NC_045512.2) proteins were retrieved from the NCBI database. The protein pairs were aligned using MAFFT v.7.511 with the G-INS-I method, using 1000 maximum iterative refinements. Then, the percentage identity of each protein pair was calculated from the alignment.

### Transcriptomic read pre-processing and quantification

Transcriptomic reads were pre-processed to ensure the quality of read alignment. First, adapter trimming and read filtering were performed using fastp v0.21.0 in the default mode.^45^ Then, the trimmed reads were mapped to the human (GCF_000001405.39), golden hamster (GCF_017639785.1), SARS-CoV-2 (NC_045512.2), or B236 (MZ937003.2) genomes using STAR aligner v2.6.1c^50^ with 5, 500,000, 1,000,000, 1, 0.33, and 0.33 as the maximum number of mismatches per pair, maximum intron length, maximum gaps between two mates, minimum overhang for annotated spliced alignments, minimum number of bases matched over read length, and minimum alignment score over read length, respectively, different from the default mode. All read samples contain >97.27% of mapped reads, indicating the lack of substantial contamination from other organisms (Supplementary Table S8). After that, the mapped reads were quantified using featureCounts provided in the subread package v.1.6.3 with 0.25 as the minimum fraction of overlapping bases.^51^

### Differential expression analysis

Differential expression and functional enrichment analyses were performed using R v4.3.1.^52^ The presence or lack of gene expression in a control and a treatment group was determined from the z standard score of fragments per kilobase million (zFPKM)^53^ using the zFPKM R package v1.22.0.^54^ In each group, a gene whose zFPKM average > −3 was considered actively expressed or having expression, whereas that whose zFPKM average ≤ −3 was considered inactive or lacking expression. Differential expression analysis was then performed using Deseq2 v1.40.2^55^ with the apeglm shrinkage estimator implemented in apeglm R package v1.22.1.^56^ For the hamster dataset, the mock-infected samples at 5 d.p.i. were used as control for the samples with the three viruses at both 2 and 5 d.p.i. In the pairwise comparison, a gene with a Benjamini-Hochberg adjusted *P* value of the Wald test <0.05 and zFPKM value in either of the two comparison groups > −3 was considered a DEG. Principal component analysis of log_2_ expression fold change profile was performed using the PCAtools R package v2.12.0.^57^ The human-hamster orthologous relationship data were collected from the NCBI Gene database^58^ on July 30, 2022 for comparing the results between species.

### Functional enrichment analysis

Genes were categorized into six groups based on the direction of gene regulation in B236 and SARS-CoV-2 infection experiments compared to the mock-infected control. The six gene categories include B236-Up/SC2-Up, B236-Down/SC2-Down, B236-Up/SC2-notUp, B236-Down/SC2-notDown, B236-notUp/SC2-Up, and B236-notDown/SC2-Down genes. Up, Down, notUp, and notDown stand for genes whose expression was upregulated, downregulated, not upregulated (downregulated or not significantly changed), and not downregulated (upregulated or not significantly changed) compared to the mock-infected group, respectively. The level of concordance or discordance in gene regulation across the two RNA sequencing experiments were expressed in an absolute value of the disco score.^59^ Only genes with a Benjamini-Hochberg adjusted *P* value of the likelihood ratio test <0.05 and zFPKM values in all comparison groups > −3 were retained for functional comparison. First, the libraries of GO and KEGG 2021 terms were collected from the Enrichr website (https://maayanlab.cloud/Enrichr/#libraries). The top 100 genes in each category with the highest absolute values of the disco scores were selected, and gene set enrichment analysis was subsequently performed using the clusterProfiler R package v4.8.1.^60^ An ontology term was considered significantly enriched if its Benjamini-Hochberg adjusted *P* value from the hypergeometric test was <0.05.

### SARS-CoV-2 and B236 S-based fusion assay

A SARS-CoV-2 and B236 S-based fusion assay (Fig. 2b and c) was performed as previously described.^18^^,^^20^^,^^21^^,^^30^^,^^31^^,^^37^^,^^38^^,^^40^^,^^41^^,^^61^^,^^62^ Briefly, on day 1, effector cells (i.e., S-expressing cells) and target cells (see below) were prepared at a density of 0.6–0.8 × 10^6^ cells in a 6-well plate. On day 2, for the preparation of effector cells, HEK293 cells were cotransfected with the S expression plasmids (400 ng) and pDSP_8-11_^63^ (400 ng) using TransIT-LT1 (Takara, Cat# MIR2300). To prepare target cells, HOS-ACE2/TMPRSS2 cells and VeroE6/TMPRSS2 cells were transfected with pDSP_1-7_ (400 ng). On day 3 (24 h posttransfection), 16,000 effector cells were detached and reseeded into a 96-well black plate (PerkinElmer, Cat# 6005225), and target cells were reseeded at a density of 1,000,000 cells/2 ml/well in 6-well plates. On day 4 (48 h posttransfection), target cells were incubated with EnduRen live cell substrate (Promega, Cat# E6481) for 3 h and then detached, and 32,000 target cells were added to a 96-well plate with effector cells. *Renilla* luciferase activity was measured at the indicated time points using Centro XS3 LB960 (Berthold Technologies). For measurement of the surface expression level of the S protein, effector cells were stained with rabbit anti-SARS-CoV-2 S S1/S2 polyclonal antibody (Thermo Fisher Scientific, Cat# PA5-112048, 1:100). Normal rabbit IgG (Southern Biotech, Cat# 0111-01, 1:100) was used as a negative control, and APC-conjugated goat anti-rabbit IgG polyclonal antibody (Jackson ImmunoResearch, Cat# 111-136-144, 1:50) was used as a secondary antibody. The surface expression level of S proteins (Fig. 2a) was measured using a FACS Canto II (BD Biosciences) and the data were analyzed using FlowJo software v10.7.1 (BD Biosciences). Gating strategy for flow cytometry is shown in Supplementary Fig. S7. For calculation of fusion activity, *Renilla* luciferase activity was normalized to the mean fluorescence intensity (MFI) of surface S proteins. The normalized value (i.e., *Renilla* luciferase activity per the surface S MFI) indicating genuine fusion activity is shown as arbitrary units.

### Plaque assay

The plaque assay was performed as previously described.^18^^,^^37^^,^^38^ In brief, one day before infection, VeroE6/TMPRSS2 cells (200,000 cells) were seeded into a 12-well plate and infected with SC2, SC2ΔFCS or B236 (1,000, 100, 10 and 1 TCID_50_) at 37 °C. At 2 h.p.i., mounting solution containing 3% FBS and 1.5% carboxymethyl cellulose (Wako, Cat# 039-01335) was overlaid, followed by incubation at 37 °C. At 3 d.p.i., the culture medium was removed, and the cells were washed with PBS three times and fixed with 4% paraformaldehyde phosphate (Nacalai Tesque, Cat# 09154-85). The fixed cells were washed with tap water, dried and stained with staining solution (0.1% methylene blue (Nacalai Tesque, Cat# 22412-14) in water) for 30 min. The stained cells were washed with tap water and dried, and the size of plaques was measured using Fiji software v2.2.0 (ImageJ).

### Animal experiments

Animal experiments (Fig. 3) were performed as previously described.^20^^,^^21^^,^^23^^,^^30^^,^^31^^,^^37^^,^^38^^,^^40^ Syrian hamsters (Slc:Syrian, male, 4 weeks old) were purchased from Japan SLC Inc. (Shizuoka, Japan). For the virus infection experiments, hamsters were anesthetized by intramuscular injection of a mixture of 0.15 mg/kg medetomidine hydrochloride (Domitor®, Nippon Zenyaku Kogyo), 2.0 mg/kg midazolam (Dormicum®, Fujifilm Wako, Cat# 135-13791) and 2.5 mg/kg butorphanol (Vetorphale®, Meiji Seika Pharma) or 0.15 mg/kg medetomidine hydrochloride, 4.0 mg/kg alphaxaone (Alfaxan®, Jurox) and 2.5 mg/kg butorphanol. SC2, SC2ΔFCS, B236 (10,000 TCID_50_ in 100 μl) or saline (100 μl) was intranasally inoculated under anesthesia. Body weight was recorded daily by 7 d.p.i and at 10 and 14 d.p.i. Enhanced pause (Penh), the ratio of time to peak expiratory follow relative to the total expiratory time (Rpef), and the frequency of breath (breaths per minutes; BPM) were measured every day until 7 d.p.i. (see below). Lung tissues were anatomically collected at 2 and 5 d.p.i. The viral RNA load in the respiratory tissues was determined by RT-qPCR. Viral titres in the lung hilum were determined by TCID_50_. These tissues were also used for IHC and histopathological analyses (see below).

### Lung function test

Lung function tests (Fig. 3a) were routinely performed as previously described.^20^^,^^21^^,^^30^^,^^31^^,^^37^^,^^38^^,^^40^ The three respiratory parameters (Penh, Rpef and BPM) were measured by using a Buxco Small Animal Whole Body Plethysmography system (DSI) according to the manufacturer's instructions. In brief, a hamster was placed in an unrestrained plethysmography chamber and allowed to acclimatize for 30 s. Then, data were acquired over a 2.5-min period by using FinePointe Station and Review software v2.9.2.12849 (DSI).

### Immunohistochemistry

Immunohistochemical (IHC) analysis (Fig. 3d) was performed as previously described^20^^,^^21^^,^^30^^,^^31^^,^^37^^,^^38^^,^^40^ using an Autostainer Link 48 (Dako). The deparaffinized sections were exposed to EnVision FLEX target retrieval solution high pH (Agilent, Cat# K8004) for 20 min at 97 °C for activation, and a mouse anti-SARS-CoV-2 N monoclonal antibody (clone 1035111, R&D Systems, Cat# MAB10474-SP, 1:400) was used as a primary antibody. The sections were sensitized using EnVision FLEX for 15 min and visualized by peroxidase-based enzymatic reaction with 3,3′-diaminobenzidine tetrahydrochloride (Dako, Cat# DM827) as substrate for 5 min. The N protein positivity was evaluated by certificated pathologists as previously described.^20^^,^^21^^,^^30^^,^^31^^,^^37^^,^^38^^,^^40^ Images were incorporated as virtual slides by NDP.scan software v3.2.4 (Hamamatsu Photonics). The N-protein positivity was measured as the area using Fiji software v2.2.0 (ImageJ).

### H&E staining

Haematoxylin and eosin (H&E) staining (Fig. 3e) was performed as previously described.^20^^,^^21^^,^^30^^,^^31^^,^^37^^,^^38^^,^^40^ Briefly, excised animal tissues were fixed with 10% formalin neutral buffer solution and processed for paraffin embedding. The paraffin blocks were sectioned at a thickness of 3 μm and then mounted on MAS-GP-coated glass slides (Matsunami Glass, Cat# S9901). H&E staining was performed according to a standard protocol.

### Histopathological features of lung lesions

The presence of inflammation area including type II pneumocytes and large type II pneumocytes (Fig. 3e), were evaluated by certified pathologists.

### Immunofluorescence assay of human iPSC-derived colon organoids

For immunofluorescence staining of human iPSC-derived colon organoids, the cells were fixed with 4% paraformaldehyde in PBS at 4 °C. Human iPSC-derived colon organoids were harvested and used for the preparation of frozen section (5 μm). The sections were permeabilized using Tris Buffered Saline with 0.1%-Tween 20 Detergent (Nacalai Tesque, Cat# 34874-91) for 10 min and blocked using Blocking One Histo (Nacalai Tesque, Cat# 06349-64) for 45 min. The sections were incubated in Tris Buffered Saline with 0.1%-Tween 20 Detergent with or without primary antibodies overnight at 4 °C. The sections were washed with PBS and incubated in Tris Buffered Saline with 0.1%-Tween 20 Detergent containing Alexa Fluor 488- or 594-conjugated secondary antibodies for 45 min. The sections were finally washed and mounted with Fluoro-KEEPER Antifade Reagent, Non-Hardening Type with DAPI (Nacalai Tesque, Cat# 12593-64) and analyzed using the BZ-X700 (Keyence Corporation). Anti-Villin antibodies (abcam, Cat# ab109516) and anti-SARS-CoV/SARS-CoV-2 Nucleocapsid antibodies (GeneTex, Cat# GTX632269) were used as primary antibodies.

### Pseudovirus and cell preparations for infection or protease experiments

Pseudoviruses for protease were prepared as previously described.^17^^,^^20^^,^^21^^,^^30^^,^^31^^,^^38^^,^^40^^,^^41^^,^^64^, ^65^, ^66^, ^67^, ^68^ Briefly, lentivirus (HIV-1)-based, luciferase-expressing reporter viruses were pseudotyped with SC2, SC2ΔFCS or B236 S proteins. HEK293T cells (1,000,000 cells) were cotransfected with 1 μg psPAX2-IN/HiBiT,^16^ 1 μg pWPI-Luc2,^16^ and 500 ng plasmids expressing S proteins using PEI Max (Polysciences, Cat# 24765-1) according to the manufacturer's protocol. Two days posttransfection, the culture supernatants were harvested and centrifuged. The pseudoviruses were stored at −80 °C until use.

HEK293-ACE2 cells^18^ transiently expressing various human proteases were prepared 1 d prior to pseudovirus infection. Briefly, HEK293-ACE2 cells (500,000 cells) were transfected with 200 ng protease plasmids using Opti-MEM (gibco, Cat#31985-070) and PEI Max (Polysciences, Cat# 24765-1) according to the manufacturer's protocol. pCAGGS empty vector^36^ was used as a vector control. The cells were incubated overnight and then seended in a 96-well white plate (10,000 cells/well). Expression plasmids of a variety of human proteases were used in the previous study.^69^ The expression plasmid of human ADAM9^70^ was kindly provided from Prof. Yuh-Pyng Sher.

For pseudovirus infection (Supplementary Fig. S3), the amount of input virus was normalized to the HiBiT value measured by Nano Glo HiBiT lytic detection system (Promega, Cat# N3040)], which indicates the amount of p24 HIV-1 antigen. For target cells, the HOS-TMPRSS2 cells stably expressing Hunan or Hamster ACE2^35^ were used. Two d.p.i., the infected cells were lysed with a Bright-Glo Luciferase Assay System (Promega, cat# E2620) and the luminescent signal was measured using a GloMax Explorer Multimode Microplate Reader (Promega).

### Protease experiments

Pseudovirus infection (Supplementary Fig. S5d–f) was performed as previously described.^17^^,^^20^^,^^21^^,^^30^^,^^31^^,^^38^^,^^40^^,^^41^^,^^64^, ^65^, ^66^, ^67^, ^68^ Briefly, diluted 100ul/well of the SC2, SC2ΔFCS and B236 S pseudoviruses (5.0 × 10^4^ relative light units) were added to HEK293-ACE2/protease expressing cells in a 96-well white plate (see “Pseudovirus and cell preparations for protease experiments” section). At 2 d.p.i., the infected cells were lysed with a Bright-Glo luciferase assay system (Promega, Cat# E2650), and the luminescent signal was measured using a GloMax explorer multimode microplate reader 3500 (Promega). The fold change of infectivity in each cell was normalized to cell transfected with empty vector control.

### Statistics

Statistical significance was tested using a two-sided Mann–Whitney U test, a two-sided Welch's test or a two-sided Student's t test unless otherwise noted. Before performing a two-sided Student's t test, we confirmed normality of observations and homogeneity of variance. Normality of observations was initially tested using the Shapiro–Wilk normality test, and when *P* values were lower than 0.05, a two-sided Mann–Whitney U test was used instead of a two-sided Student's t test. Next, homogeneity of variance was tested using F test, and when *P* values were equal or larger than 0.05, a two-sided Welch's test was used instead of a two-sided Student's t test. The tests above were performed using Prism 9 software v9.1.1 (GraphPad Software).

In the time-course experiments (Fig. 1, Fig. 2b and d, 3a–c, 4a and b), we performed a permutation test using the area under the curve (AUC) as a summary measure. We first calculated the observed mean AUC across time points for each condition (e.g., Virus A vs. Virus B). We then randomized the condition labels at each time point and computed the mean AUC for these permuted datasets. This permutation process was repeated 10,000 times to generate a distribution of mean AUC values under the null hypothesis that the condition has no effect. By comparing the observed mean AUC with the distribution of permuted mean AUC values, we calculated an empirical *P*-value for the difference observed between conditions. Subsequently, familywise error rates (FWERs) were calculated by the Holm method. The computational code supporting this analysis is publicly available in our GitHub repository (https://github.com/TheSatoLab/B236_full). These analyses were performed in R v4.1.2 (https://www.r-project.org/).

All statistical tests used in viral count and expression fold change comparison were performed using the rstatix R package v0.7.2.^71^ Normality and homogeneity of variances of a dataset were initially tested using the Shapiro–Wilk and Levene's tests, respectively, before performing multiple group comparisons. The type I error rate was set to 0.05 for both tests. One-way ANOVA, Welch's ANOVA, or Kruskal–Wallis tests were chosen for hypothesis testing, followed by Tukey's HSD, Games–Howell, or Dunn's post hoc tests, respectively, based on the result of tests for normality and homogeneity of variances. Benjamini-Hochberg correction was applied for one-way ANOVA and Kruskal–Wallis tests, but not Welch's ANOVA test which Welch's correction was implemented implicitly.

### Role of funders

The funders had no role in the study design, data collection, analysis, interpretation, the writing of this report, or any aspect pertinent to this study.

## Results

### Growth kinetics of B236 in cell cultures

**Supplementary Fig. S1:**
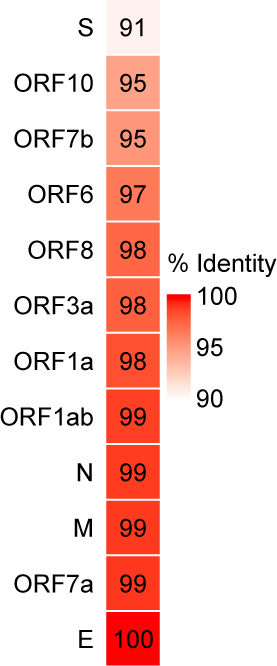
**Percentage identity between B236 and SARS-CoV-2 proteins**.

To investigate the virological features of B236, we prepared the B236 isolate.^15^ We also prepared a lineage of original SARS-CoV-2 (strain WK-521, PANGO lineage A).^19^ The S, ORF10, and ORF7b proteins exhibit more substantial differences between SARS-CoV-2 and B236 compared to other proteins (Supplementary Fig. S1). As described in the introduction, the S protein of B236 lacks the FCS, a hallmark of SARS-CoV-2.^15^ To address the impact of FCS on virological properties, we artificially generated SC2ΔFCS by reverse genetics.^39^ Western blotting analysis of infected VeroE6/TMPRSS2 cells verified that the S protein of SARS-CoV-2 was cleaved into two subunits, while those of SC2ΔFCS and B236 were not (Fig. 1a).

We then inoculated these three viruses into five cell cultures: Vero cells (Fig. 1b), VeroE6/TMPRSS2 cells (Fig. 1c), AO-ALI system (Fig. 1d), HiAlv (Fig. 1e), and HiTrach (Fig. 1f). In Fig. 1b–e, the growth of B236 was marginally lower than that of SARS-CoV-2. The growth of SC2ΔFCS was comparable to that of the parental SARS-CoV-2 in these four cultures (Fig. 1b–e).

Compared to these four cell culture systems (Fig. 1b–e), the growth of B236 in HiTrach was prominently lower than that of SARS-CoV-2 (FWER = 0.00030) (Fig. 1f). Because the growth of B236 was lower than that of SC2ΔFCS in HiTrach (Fig. 1f), the attenuated growth of B236 compared to that of SARS-CoV-2 in HiTrach is not only due to the absence of FCS in S protein.

**Supplementary Fig. S2:**
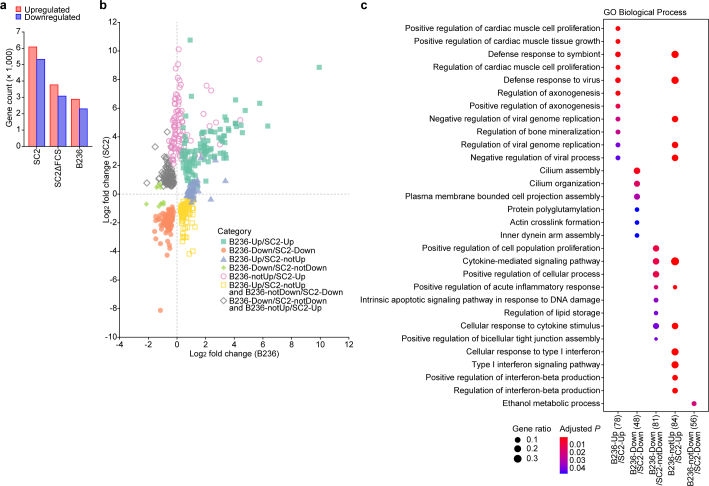
**Functional enrichment analysis of the B236- and SARS-CoV-2-infected airway epithelial cells.** (**a**) Number of upregulated and downregulated differentially expressed genes (DEGs), genes whose expression differs among comparison groups, in the human iPSC-derived airway epithelial cells (HiTrach) infected with SARS-CoV-2 (SC2), FCS-deleted SARS-CoV-2 (SC2ΔFCS), and BANAL-20-236 (B236). (**b**) Scatter plot showing log_2_ fold change of the top 100 genes with the highest disco scores^54^ whose expression was upregulated or downregulated in the B236- and SC2-infected airway epithelial cells. B236-Up/SC2-Up, B236-Down/SC2-Down, B236-Up/SC2-notUp, B236-Down/SC2-notDown, B236-notUp/SC2-Up, and B236-notDown/SC2-Down denote gene groups with different expression regulation patterns. Up, Down, notUp, and notDown stand for genes whose expression was upregulated, downregulated, not upregulated (downregulated or not significantly changed), and not downregulated (upregulated or not significantly changed) compared to the mock-infected group, respectively. (**c**) Functional enrichment plot showing gene ratio and adjusted *P* value of the top ten GO Biological Process terms enriched with the top 100 genes of each expression category shown in (**b**). The number in parentheses represents the number of identified genes in the GO Biological Process database.

To investigate the difference in transcriptome of human airway epithelial cells in response to B236, SARS-CoV-2, and SC2ΔFCS infection, RNA sequencing (RNA-Seq) analysis was conducted using the infected HiTrach cultures (Fig. 1f) at 72 h postinfection (h.p.i.). Consistent with that in the suspension of apical cell surface measured by RT-qPCR (Fig. 1f, top), viral RNA amount in the B236-infected cells was lower than SARS-CoV-2-infected and SC2ΔFCS-infected cells (Fig. 1g). The comparative functional enrichment analysis showed that transcriptome alteration by B236 infection was different from those by SARS-CoV-2 and SC2ΔFCS infections (Supplementary Fig. S2a–c and Supplementary Tables S1 and S2). Particularly, the expression of interferon-stimulating genes (ISGs), several of which are related to the suppression of SARS-CoV-2 replication,^24^, ^25^, ^26^, ^27^, ^28^ was robustly induced in the SARS-CoV-2-infected and SC2ΔFCS-infected cells (adjusted *P* < 0.0001 by Dunn test) but not in the B236-infected cells (Fig. 1h and i). These differences in gene regulation are likely attributed to the lower replication of B236. Altogether, our results suggest that B236 is less replicative in airway epithelial cells than SARS-CoV-2 and SC2ΔFCS.

### Fusogenicity of B236

Our previous studies on the SARS-CoV-2 VOCs suggested that the cleavage efficiency of S protein is associated with the fusogenicity of S protein.^37^^,^^38^^,^^62^ For instance, compared to the ancestral SARS-CoV-2 S, the Delta S is well cleaved and more fusogenic, while the Omicron S is less cleaved and less fusogenic.^37^^,^^38^^,^^62^ Because the B236 S is not cleaved (Fig. 1a), we hypothesized that B236 is faintly fusogenic. To address this possibility, we first conducted the cell-based fusion assay.^61^ Flow cytometry showed that the surface expression level of B236 S was significantly lower than those of SARS-CoV-2 and SC2ΔFCS S proteins (*P <* 0.0001 by Student's *t* test, Fig. 2a). As expected, the cell-based fusion assay showed that the S protein of SC2ΔFCS is not fusogenic compared to that of the SARS-CoV-2 S (Fig. 2b). Similarly, the B236 S had little or no fusogenicity (Fig. 2b). Second, we performed a plaque assay using live viruses and VeroE6/TMPRSS2 cells. Consistent with the cell-based fusion assay (Fig. 2b), the plaque size of B236-infected cells was significantly smaller than that of SC2-infected cells (1.4-fold, *P =* 0.0023 by Mann–Whitney U test) (Fig. 2c). Third, to quantify the invasive capacity of B236 on the airway epithelial-endothelial barriers, which mirrors viral fusogenicity, the number of viruses that invaded the barrier from the top channel (Fig. 2d, left) to the bottom channel (Fig. 2d, right) of an airway-on-a-chip system was measured.^20^, ^21^, ^22^, ^23^^,^^30^ We used Delta and Omicron BA.1, the SARS-CoV-2 VOCs, as positive and negative controls, respectively (Fig. 2d).^37^^,^^38^ The percentage of B236 that invaded to the bottom channel of the airway-on-a-chip system was significantly lower than that of SARS-CoV-2 (*P <* 0.0001 by Student's *t* test; Fig. 2e). Moreover, the percentage of B236 invaded to the bottom channel was comparable to that of Omicron BA.1, the negative control of the assay (Fig. 2e). Altogether, these results suggest that B236 exhibits little or no fusogenicity.

### Virological features of B236 *in vivo*

To investigate the pathogenicity of B236 *in vivo*, B236 as well as SARS-CoV-2 and SC2ΔFCS were intranasally inoculated into hamsters. SARS-CoV-2-infected hamsters exhibited decreased body weight, Rpef, and BPM and increased Penh (Fig. 3a). On the other hand, although these four values of SC2ΔFCS-infected hamsters were significantly different from those of uninfected hamsters, the extent of disease in SC2ΔFCS group was relatively milder than that of SARS-CoV-2 group. For instance, consistent with a previous study,^13^ the loss of body weight of SC2ΔFCS-infected hamsters was milder than that of SARS-CoV-2-infected hamsters with a statistical significance (FWER = 0.0006; Fig. 3a). The differences of three respiratory parameters (Penh, Rpef, and BPM) in SC2ΔFCS-infected hamsters were also relatively less than those in SARS-CoV-2-infected hamsters (Fig. 3a). In the case of B236, however, the body weight, Penh and BPM of B236-infected hamsters were significantly different from those of SC2ΔFCS-infected hamsters (body weight, FWER = 0.0006; Penh, FWER = 0.0014; BPM, FWER = 0.0006; Fig. 3a). Moreover, the body weight and BPM of B236-infected hamsters were even comparable to that of uninfected hamsters (Fig. 3a). These observations suggest that B236 is less pathogenic than SARS-CoV-2 and even than SC2ΔFCS in hamsters.

To assess the replication of the viruses inoculated, the lungs of infected hamsters were collected at 2 and 5 d.p.i. and then were separated into hilum and periphery regions. In both the lung regions, the viral RNA loads of B236-infected hamsters was significantly lower than those of SARS-CoV-2-infected and SC2ΔFCS-infected hamsters (Fig. 3b). The viral titer in the lung periphery of B236-infected hamsters was significantly lower than those of SARS-CoV-2-infected and SC2ΔFCS-infected hamsters (Fig. 3c). Particularly, at 5 d.p.i., the viral RNA loads of B236-infected hamsters sharply decreased (lung hilum, 3300-fold; and lung periphery, 290-fold) (Fig. 3b). Furthermore, the TCID_50_ of B236 was undetectable at 5 d.p.i (Fig. 3c).

**Supplementary Fig. S3:**
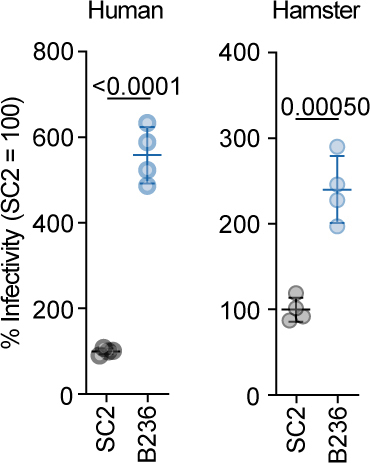
**Pseudovirus assay in human and hamster ACE2 expressing cells.** HIV-1-based reporter viruses pseudotyped with the S proteins of B236 or SARS-CoV-2 were prepared. The pseudoviruses were inoculated into a series of HOS-TMPRSS2 cells stably expressing human or hamster ACE2 cells at 1 ng HIV-1 p24 antigen. The percent infectivity compared with that of the virus pseudotyped with the SARS-CoV-2 (SC2) protein are shown. Assays were performed in quadruplicate. The presented data are expressed as the average ± standard deviation (SD). Statistically significant differences were determined by a two-sided Student's t test.

Regarding the low viral propagation in B236-infected hamsters, it is possible that the affinity of B236 S to hamster ACE2 is lower than that of SARS-CoV-2 S. To address this possibility, we prepared the lentivirus-based pseudoviruses harboring the spike proteins of SARS-CoV-2 and B236. The infection experiments using the pseudoviruses and the cells expressing human and hamster ACE2 showed that the pseudovirus with B236 S exhibits significantly higher infectivity than that with SARS-CoV-2 S in both the cells expressing human and hamster ACE2 (Supplementary Fig. S3). These results suggest that the attenuated growth of B236 in hamsters (Fig. 3b and c) is not due to the decreased affinity of B236 S to hamster ACE2.

Next, we investigated the pathogenicity of B236 by analyzing a formalin-fixed right lung of the infected hamsters. We carefully identified the main bronchus, four lobes of the right lung, and lobar bronchi and sectioned each lobe along with bronchial branches as previously described.^20^^,^^30^^,^^38^ We stained viral nucleocapsid (N) protein in the whole lung lobes to investigate virus spread in the lung of infected hamsters (Fig. 3d). The number of N-positive cells in the SC2ΔFCS-infected lungs was significantly lower than that in the SARS-CoV-2-infected lungs at 2 and 5 d.p.i. (*P =* 0.029 for 2 d.p.i., *P =* 0.029 for 5 d.p.i. by Mann–Whitney U test). Moreover, at 5 d.p.i, the number of N-positive cells in B236-infected lungs was lower than that in SC2ΔFCS-infected lungs and was almost undetectable (*P* = 0.029 by Mann–Whitney U test).

To analyze the degree of lung inflammation, the total inflammation area including the area of hyperplastic large type II pneumocytes was measured at 2 and 5 d.p.i. (Fig. 3e). The percentages of the area of hyperplastic large type II pneumocytes of the hamsters infected with B236 (*P =* 0.029 for 2 d.p.i., *P =* 0.029 for 5 d.p.i. by Mann–Whitney U test) and SC2ΔFCS (*P =* 0.029 for 2 d.p.i., *P* = 0.029 for 5 d.p.i. by Mann–Whitney U test) were significantly lower than that of SARS-CoV-2-infected hamsters (Fig. 3e). These results suggest that the intrinsic pathogenicity of B236 is lower than that of SARS-CoV-2.

Next, we performed RNA-Seq analysis to investigate the difference in the transcriptome of hamster lungs in response to these three viruses. At 2 d.p.i., viral RNA amounts in the B236-infected lungs were lower than those of the SARS-CoV-2-infected lungs but comparable to those of the SC2ΔFCS-infected lungs (Fig. 3f). However, at 5 d.p.i., viral RNA amounts in the B236-infected lungs were lower than those in the SARS-CoV-2-infected and SC2ΔFCS-infected lungs (Fig. 3f). These results suggest that B236 can infect hamster lung, and it is cleared more rapidly compared to SARS-CoV-2 and SC2ΔFCS. Similarly, at 2 d.p.i., the numbers of differentially expressed genes (DEGs), genes whose expression differs among comparison groups, in the B236-infected lungs were lower than those in the SARS-CoV-2-infected lungs but comparable to or even higher than those in the SC2ΔFCS-infected lungs (Fig. 3g). Contrastingly, at 5 d.p.i., the numbers of DEGs in the B236-infected lungs were lower than those in the SARS-CoV-2-infected and SC2ΔFCS-infected lungs (Fig. 3g). These results suggest the impact of viral infection on the lung transcriptome more rapidly decreases in the B236-infected hamsters compared to the SARS-CoV-2-infected and SC2ΔFCS-infected hamsters.

**Supplementary Fig. S4:**
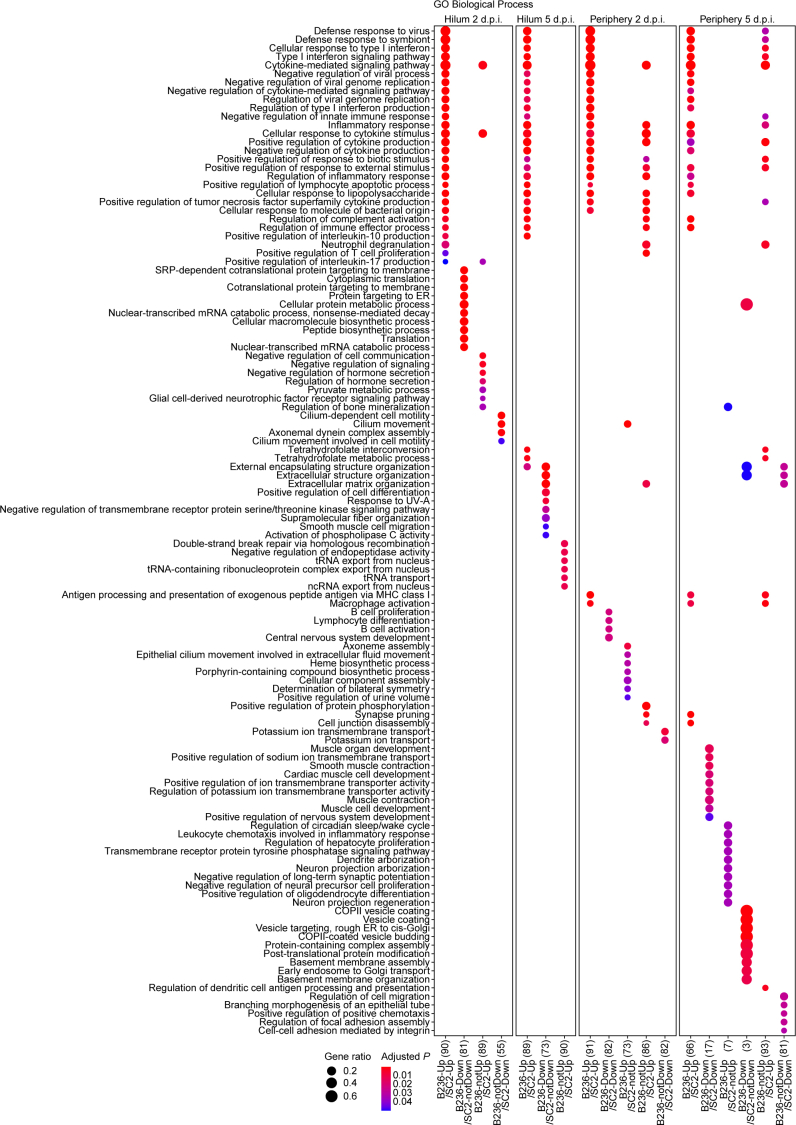
**Functional enrichment analysis of the B236- and SARS-CoV-2-infected hamster lung hila and peripheries at 2 and 5 d.p.i.** Functional enrichment plot showing gene ratio and adjusted *P* value of the top ten GO Biological Process terms enriched with the top 100 genes with the highest disco scores in each expression category. See "Functional enrichment analysis” section for denomination of the gene expression categories in detail. The number in parentheses represents the number of identified genes in the GO Biological Process database.

Although the expression of genes related to innate immune response was robustly upregulated in the hamster lungs infected with the three viruses at 2 d.p.i., the expression of some of these genes, such as *IFIH1*, *IFNB1*, and *STAT2*, was less induced in the B236-infected lungs at 5 d.p.i. (Fig. 3h and i, Supplementary Fig. S4, and Supplementary Tables S3 and S4). This is probably due to the greater decrease in the amount of B236 compared to that of SARS-CoV-2 and SC2ΔFCS (Fig. 3f). Altogether, our results suggest that B236 is less pathogenic than SARS-CoV-2 and SC2ΔFCS, probably because of the faster clearance of B236.

### Enterotropism of B236

**Supplementary Fig. S5:**
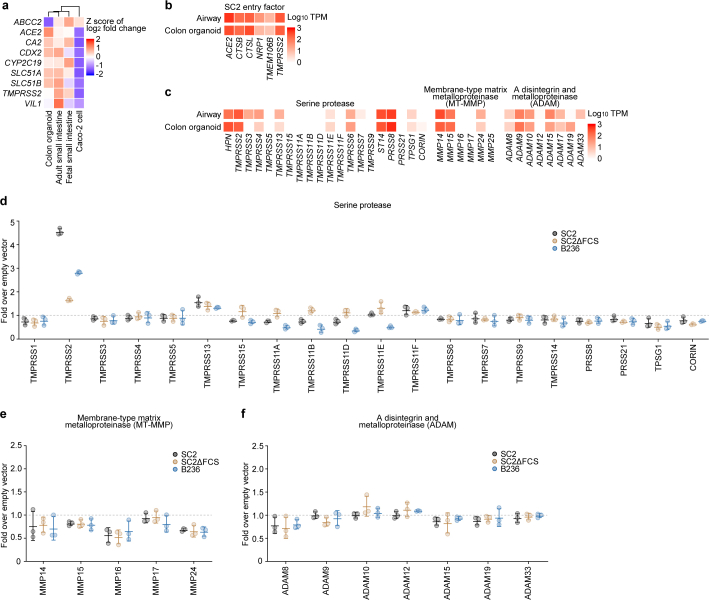
**Search for human proteases involved in SARS-CoV-2 and B236 infection.** (**a**) The z score of log_2_ expression fold change of intestinal marker genes in the human iPSC-derived colon organoids, total RNA of human adult and fetal small intestines, and Caco-2 cells. The dendrogram was depicted using hierarchical clustering with Ward’s method. (**b**) Normalized count of six known SARS-CoV-2 entry factor genes^73^, ^74^, ^75^, ^76^ in the mock-infected airway epithelial cell cultures and colon organoids in transcripts per million (TPM) unit. (**c**) Normalized count of 34 human proteases, including 20 serine proteases, 6 membrane-type matrix metalloproteinases (MMPs), and 8 a disintegrin and metalloproteinases (ADAMs), in the mock-infected airway epithelial cells and colon organoids in transcripts per million (TPM) unit. (**d–f**) Pseudovirus infection assay with protease expression. HIV-1-based reporter viruses pseudotyped with the S proteins of SARS-CoV-2 (SC2), FCS-deleted SARS-CoV-2 (SC2ΔFCS) and BANAL-20-236 (B236) were prepared. Pseudovirus infection (1.5×10^6^ relative light unit each virus) was performed in HEK293 cells overexpressing human ACE2 and **(d)** serine proteases, **(e)** MMPs or **(f)** ADAMs. The fold change of infectivity in each cell was normalized to cells transfected with empty vector control.

Because B236 and several bat SC2r-CoVs were isolated from feces or rectal swabs,^7^, ^8^, ^9^, ^10^, ^11^^,^^15^ it is hypothesized these bat SC2r-CoVs, including B236, infect intestinal tissues and replicate. To address this possibility, we prepared human iPSC-derived colon organoids.^72^ We validated that the expression profiles of the marker genes of human intestines in the human iPSC-derived colon organoids were similar to those of adult small intestines rather than Caco-2 cells, a human colon cell line (Supplementary Fig. S5a). Then, the human colon organoids were infected with B236, SARS-CoV-2, and SC2ΔFCS. Consistent with the experimental results using the human respiratory cultures (Fig. 1d–f), the growth kinetics of SARS-CoV-2 and SC2ΔFCS was comparable (Fig. 4a). Interestingly, although the growth of B236 is lower than that of SARS-CoV-2 in HiTrach (Fig. 1f), the growth of B236 was significantly greater than that of SARS-CoV-2 in human colon organoids (FWER = 0.00030) (Fig. 4a). Because the growth kinetics of B236 was comparable to those of SARS-CoV-2 and SC2ΔFCS in the human colon Caco-2 cell line (Fig. 4b), our data suggest that the growth advantage of B236 can be observed only in the human iPSC-derived colon organoids. As shown in Fig. 4c, a larger number of N-positive cells was detected in the colon organoids infected with B236 than that with SARS-CoV-2. These results suggest that B236 is more enterotropic compared to SARS-CoV-2.

Next, we performed RNA-Seq analysis using the infected colon organoids at 72 h.p.i. Viral RNA amount in the B236-infected colon organoid was significantly higher than that in the SARS-CoV-2-infected and SC2ΔFCS-infected organoids (adjusted *P* < 0.0001 by Tukey's HSD test) (Fig. 4d). The number of DEGs in B236-infected colon organoids was higher than those in SARS-CoV-2-infected and SC2ΔFCS-infected colon organoids (Fig. 4e and Supplementary Table S6), suggesting that B236 infection has a larger impact on cellular transcriptome than SARS-CoV-2 and SC2ΔFCS infection in colon organoids. Furthermore, the principal component (PC) analysis suggests that the pattern of transcriptome alteration caused by B236 infection in colon organoids (represented by PC1, explaining 57.69% of the total variance) is clearly different from those caused by SARS-CoV-2 and SC2ΔFCS infections in airway epithelial cells (represented by PC2, explaining 28.49% of the total variance) (Fig. 4f).

**Supplementary Fig. S6:**
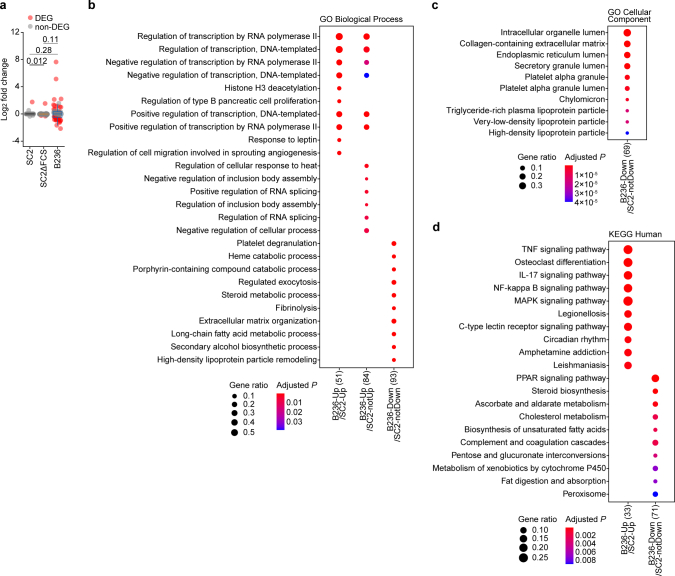
**Functional enrichment analysis of the B236- and SARS-CoV-2-infected colon organoids.** (**a**) The log_2_ expression fold change of genes listed in the GO Biological Process term “cellular response to type I interferon” (GO:0071357) in the colon organoids infected with SC2, SC2ΔFCS, and B236. Each circle indicates a data point. Red circle, DEG; grey circle, non-DEG. Numbers above the box plots are adjusted *P* values calculated by Dunn test. (**b–d**) Functional enrichment plots showing gene ratios and adjusted *P* values of the top ten (**b**) GO Biological Process, (**c**) GO Cellular Component, and (**d**) KEGG terms enriched with the top 100 genes with the highest disco scores in each expression category. See “Functional enrichment analysis” section for denomination of the gene expression categories in detail. The number in parentheses represents the number of identified genes in each functional database.

**Supplementary Fig. S7:**
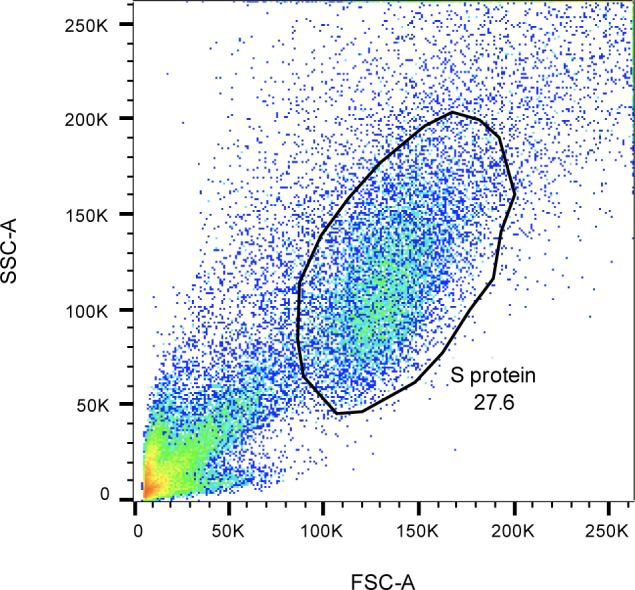
**Gating strategy for flow cytometry.** A representative dot plot of the gating for flow cytometry is shown. FSC, forward scatter; SSC, side scatter.

It is possible that the difference in tissue tropism of B236 and SARS-CoV-2 between airway respiratory cells (Fig. 4f) and colon organoids (Fig. 4a, c, and 4d) is resulted from the different expression levels of entry factors genes, including *ACE2*, *TMPRSS2*,^73^ *CTSB*, *CTSL*,^74^ *NRP1*,^75^ and *TMEM106B*.^76^ To address this possibility, we used our RNA-Seq dataset and compared the expression level of these genes related to viral entry. As shown in Supplementary Fig. S6b, however, the expression levels of these genes were comparable between human airway epithelial cells and colon organoids, suggesting that the different tissue tropism of B236 and SARS-CoV-2 is not explained by the different levels of entry factors.

Additionally, we further investigated whether specific cellular proteases, other than TMPRSS2, expressed in the colon, could support B236 infection but not SARS-CoV-2 infection. In fact, a recent study showed that the membrane-type matrix metalloproteinases enhance the infection efficiency of the SARS-CoV-2 Omicron variant but not of ancestral SARS-CoV-2.^69^ Some protease genes, such as *PRSS8*, *TMPRSS1*, *TMPRSS14*, *MMP14*, *TMPRSS2*, and *MMP15*, were highly expressed in the colon organoids than in human respiratory cells (log_10_ transcripts per million >2; Supplementary Fig. S5c). However, no protease tested promoted the infection of B236 except for TMPRSS2 (Supplementary Fig. S5d–f).

Although the expression of ISGs, including those related to the suppression of SARS-CoV-2 replication,^24^, ^25^, ^26^, ^27^, ^28^ was upregulated in the SARS-CoV-2-infected airway epithelial cells (Fig. 1h and i, and Supplementary Fig. S2c), the expression of most of these genes was not upregulated in the SARS-CoV-2-infected colon organoids (Fig. 4g, Supplementary Fig. S6a–d, and Supplementary Tables S5 and S6). The comparative functional enrichment analysis showed that the genes related to the metabolism function of colonocytes (e.g., “steroid metabolic process” and “long-chain fatty acid metabolic process”) were downregulated specifically in the B236-infected colon organoids (Supplementary Fig. S6b–d, Supplementary Tables S5 and S6). These results raise a possibility that colonocytes were particularly targeted and damaged by B236 infection. To address this possibility, we investigated for specific cells in the colonic epithelium potentially susceptible to B236 infection. The expression of marker genes for colonocytes, such as *SI*, *SLC51B*, *SLC51A*, and *VIL1*, significantly decreased in the B236-infected organoids (Fig. 4h). On the other hand, the expression of goblet cell marker genes (*MUC2* and *TFF3*) did not decrease in the B236-infected colon organoids (Fig. 4h). Furthermore, we investigated the cells in colonic epithelium susceptible to B236 infection and stained Villin, a marker of colonocyte, and viral N protein in infected colon organoids. Both SARS-CoV-2-infected and B236-infected colon organoids showed the presence of N in Villin-positive cells. Particularly, the intensity of N in Villin-positive cells in the B236-infected colon organoids was higher than that in SARS-CoV-2-infected colon organoids (Fig. 4i). Altogether, our results suggest that B236 is more enterotropic than SARS-CoV-2, and B236 targets colonocytes rather than the other cells in colonic epithelium.

## Discussion

Elucidating the mechanism of SC2r-CoV spillover from the wild is important for understanding the emergence of SARS-CoV-2 in humans and preparing for future outbreak of as-yet-unidentified coronaviruses with potentially higher risk to global health. In this study, we investigated the virological properties of B236 and compared them with those of SARS-CoV-2. We showed that B236 was less fusogenic and pathogenic than SARS-CoV-2. Although B236 replicated in human respiratory cells less efficiently than SARS-CoV-2, B236 replicated in colon organoids more efficiently than SARS-CoV-2. These data suggest that B236 has distinguishable virological features compared to SARS-CoV-2.

Our previous studies on the emerging SARS-CoV-2 VOCs, including the Delta,^38^ Omicron BA.1,^37^ BA.2^40^ and BA.5^31^ variants, suggest that the fusogenicity of S protein is closely associated with viral pathogenicity in hamsters. For instance, compared to the ancestral SARS-CoV-2, the Delta variant is more fusogenic and pathogenic, while the Omicron BA.1 variant is less fusogenic and pathogenic.^37^^,^^38^ Compared to the ancestral SARS-CoV-2, here we showed that B236 is less fusogenic in the cell cultures (Fig. 2) and less pathogenic in hamsters (Fig. 3). These observations on B236 are consistent with our hypothesis, suggesting that viral fusogenicity is a critical factor that determines viral pathogenicity not only of SARS-CoV-2 but also of SC2r-CoVs. The poorer fusogenicity and milder pathogenicity of B236 compared to those of SARS-CoV-2 are likely attributed to the absence of FCS in the B236 S protein. The milder pathogenicity due to the lack of FCS in the B236 S is also supported by previous studies using FCS-deficient SARS-CoV-2^13^ and MpCoV-GX,^77^^,^^78^ an SC2r-CoV isolated from pangolin, both of which did not exhibit obvious pathogenicity *in vivo*. These observations suggest that the pathogenicity of SARS-CoV-2 and SC2r-CoVs is closely associated with the presence of FCS in their S proteins.

As a unique feature observed only in B236-infected hamsters, we observed the data suggesting that B236 is more rapidly cleared from the lungs of infected hamsters. For instance, the viral load of B236-infected hamster deceased more steeply than those of SARS-CoV-2-infected and SC2ΔFCS-infected hamsters (Fig. 3b and c). A reduced lung tropism of B236 compared to SARS-CoV-2 has also been described in human ACE2 transgenic mice.^79^ There are at least two possible explanations for this observation: 1) viral growth of B236 in hamster lung is lower than those of SARS-CoV-2 and SC2ΔFCS; and/or 2) B236 exhibits weaker resistance to antiviral immune response than SARS-CoV-2 and SC2ΔFCS. Regarding the former scenario, the number of N-positive cells in the B236-infected lungs was lower than that of SARS-CoV-2-infected and SC2ΔFCS-infected lungs at 2 and 5 d.p.i (Fig. 3d). Also, the growth of B236 in human airway respiratory cells was significantly lower than that of SC2ΔFCS (Fig. 1f). Therefore, compared to SARS-CoV-2 and SC2ΔFCS, the reduced replication capacity of B236 in respiratory tissues can partly explain the rapid clearance of B236 (Fig. 3b and c). Regarding the latter possibility, in both the hilum and periphery of the lung of infected hamsters, the expression level of ISGs was comparable in three infection groups at 2 d.p.i. (Fig. 3h). This observation suggests that the extent of antiviral innate immune responses is comparable among these three infection groups at 2 d.p.i., but B236 was more sensitive to the induced antiviral immune responses than SARS-CoV-2 and SC2ΔFCS. Moreover, B236- and SC2ΔFCS-infected hamsters showed different patterns of the virus clearance in the lungs (Fig. 3b and c), suggesting the presence of additional factors beyond FCS that prolong viral spread in respiratory tissues. Such factors may be the proteins highly differing in amino acid sequence (Supplementary Fig. S1). Although these factors are yet unidentified, they can potentially distinguish the virological features between B236 and SARS-CoV-2.

We demonstrated that, compared to SARS-CoV-2, B236 exhibited a tropism toward intestinal cells rather than respiratory cells (Fig. 1, Fig. 4f). Our experiments using human colon organoids also suggest that B236 mainly replicated in colonocytes, which predominate in colonic epithelium, more efficiently than SARS-CoV-2. Interestingly, Caco-2 cells, a human colon cell line, did not reflect the observations in human colon organoids (Fig. 4b). These results suggest that the factor(s), which are responsible for the observed difference in viral characteristics, are specific for the human iPSC-derived colon organoids (Supplementary Fig. 5a). Our results are reminiscent of the previous reports showing that B236 is mainly enterotropic in macaques^79^ and all bat SC2r-CoVs, including B236, were identified from bat feces or rectal swabs.^7^, ^8^, ^9^, ^10^, ^11^^,^^15^ Altogether, it would be reasonable to assume that SC2r-CoVs replicate in the intestinal tissues of horseshoe bats rather than respiratory tissues.

In summary, we showed the virological properties of B236 and compared them with those of SARS-CoV-2 in human cells or tissues. Showing new insights of the virological features of SC2r-CoVs including B236, is crucial to understand the molecular mechanisms of viral spillover events and will be useful to prepare for the future pandemic possibly caused by coronaviruses.

### Strengths

We investigated the viral characteristics of SARS-CoV-2, SC2ΔFCS and B236 using not only conventional cell lines but also human respiratory epithelial cells and human colon organoids, which are more closely relevant to human organs. We revealed that B236 exhibited the tropism to human intestinal tissues. Because the deletion of FCS in SARS-CoV-2 did not change viral tissue tropism, our data suggest that FCS, the hallmark of SARS-CoV-2, cannot solely explain the difference between SARS-CoV-2 and bat SC2r-CoVs including B236.

### Limitations

The cell lines derived from the host of B236, *Rhinolophus marshalli*, have not been established, and the ACE2 sequence of *Rhinolophus marshalli* is not publicly available. Therefore, the growth efficiency of B236 and SARS-CoV-2 in the cells derived from *Rhinolophus marshalli* and/or the cells expressing *Rhinolophus marshalli* ACE2 were not tested in this study.

## Contributors

Shigeru Fujita, Sayaka Deguchi, Nasser Hesham, Izumi Kimura, Rina Hashimoto, Keiya Uriu, Daichi Yamasoba, Ziyi Guo, Alfredo A Hinay Jr., Yusuke Kosugi, Luo Chen, Lin Pan, Yu Kaku, Terumasa Ikeda, and Kazuo Takayama conducted *in vitro* experiments.

Hayato Ito, Naganori Nao, Tomokazu Tamura, Rigel Suzuki, Saori Suzuki, Izumi Kida, Keita Matsuno and Takasuke Fukuhara conducted *in vivo* experiments.

Lei Wang, Masumi Tsuda, Yoshitaka Oda, and Shinya Tanaka performed histopathological analysis.

Sayaka Deguchi, Rina Hashimoto, and Kazuo Takayama prepared airway-on-a-chip systems, AO-ALI, and iPSC-derived colon organoids.

Yuki Yamamoto and Tetsuharu Nagamoto performed generation and provision of human iPSC-derived airway and alveolar epithelial cells.

Hiroyuki Asakura, Mami Nagashima, Kenji Sadamasu and Kazuhisa Yoshimura performed viral genome sequencing analysis.

Yukie Kashima and Yutaka Suzuki performed RNA-seq analysis.

Arnon Plianchaisuk and Yukio Watanabe performed transcriptome analysis and statistical tests.

Hin Chu, Flora Donati, Sarah Temmam and Marc Eloit provided experimental materials.

Kei Sato designed the experiments and interpreted the results.

Kei Sato, Arnon Plianchaisuk, and Shigeru Fujita wrote the original manuscript.

Kei Sato and Jumpei Ito supervised the research.

The Genotype to Phenotype Japan (G2P-Japan) Consortium contributed to the project administration.

Shigeru Fujita and Arnon Plianchaisuk verified the underlying data.

All authors reviewed, proofread, and approved the final version of the manuscript.

## Data sharing statement

All transcriptomic data from human iPSC-derived airway respiratory cells, hamster lung hila and peripheries, and iPSC-derived colon organoids are available from Gene Expression Omnibus repository (GSE233943 and GSE239893).

The computational codes used in this study are available in the GitHub repository (https://github.com/TheSatoLab/B236_full).

## Declaration of interests

Yuki Yamamoto and Tetsuharu Nagamoto are founders and shareholders of HiLung, Inc. Yuki Yamamoto is a co-inventor of patents (PCT/JP2016/057254; "Method for inducing differentiation of alveolar epithelial cells", PCT/JP2016/059786, "Method of producing airway epithelial cells"). Jumpei Ito has consulting fees and honoraria for lectures from Takeda Pharmaceutical Co. Ltd. Kei Sato has consulting fees from Moderna Japan Co., Ltd. and Takeda Pharmaceutical Co. Ltd. and honoraria for lectures from Gilead Sciences, Inc., Moderna Japan Co., Ltd., and Shionogi & Co., Ltd. The other authors declare that no competing interests exist.

## Appendix A Supplementary data

Supplementary Table S8

Supplemental consortia list
