# Supplementary material for: Virological characteristics of a SARS-CoV-2-related bat coronavirus, BANAL-20-236
Source: eBioMedicine. 2024 Jun 4;104:105181. doi: 10.1016/j.ebiom.2024.105181 (PMC11215962; doi:10.1016/j.ebiom.2024.105181)
Supplement: Supplemental consortia list [file mmc9.docx]

**Consortia**

The Genotype to Phenotype Japan (G2P-Japan) Consortium

| **First names** | **Middle name** | **Surnames** | **Affiriation** |
| --- | --- | --- | --- |
| Hirofumi |  | Sawa | ^31^ |
| Keita |  | Mizuma | ^13^ |
| Jingshu |  | Li | ^13^ |
| Yume |  | Mimura | ^13^ |
| Yuma |  | Ohari | ^13^ |
| Tomoya |  | Tsubo | ^31^ |
| Zannatul |  | Ferdous | ^8^ |
| Kenji |  | Shishido | ^8^ |
| Hiromi |  | Mohri | ^8^ |
| Miki |  | Iida | ^8^ |
| Shuhei |  | Tsujino | ^4^ |
| Naoko |  | Misawa | ^1^ |
| Kaoru |  | Usui | ^1^ |
| Wilaiporn |  | Saikruang | ^1^ |
| Spyridon |  | Lytras | ^1^ |
| Shusuke |  | Kawakubo | ^1^ |
| Luca |  | Nishumura | ^1^ |
| Jarel Elgin | Mendoza | Tolentino | ^1^ |
| Wenye |  | Li | ^1^ |
| Maximilian | Stanley | Yo | ^1^ |
| Kio |  | Horinaka | ^1^ |
| Mai |  | Suganami | ^1^ |
| Mika |  | Chiba | ^1^ |
| Ryo |  | Yoshimura | ^1^ |
| Kyoko |  | Yasuda | ^1^ |
| Keiko |  | Iida | ^1^ |
| Adam | Patrick | Strange | ^1^ |
| Naomi |  | Ohsumi | ^1^ |
| Shiho |  | Tanaka | ^1^ |
| Eiko |  | Ogawa | ^1^ |
| Kaho |  | Okumura | ^1^ |
| Tsuki |  | Fukuda | ^1^ |
| Rina |  | Osujo | ^1^ |
| Isao |  | Yoshida | ^21^ |
| So |  | Nakagawa | ^32^ |
| Akifumi |  | Takaori-Kondo | ^33^ |
| Kotaro |  | Shirakawa | ^33^ |
| Kayoko |  | Nagata | ^33^ |
| Ryosuke |  | Nomura | ^33^ |
| Yoshihito |  | Horisawa | ^33^ |
| Yusuke |  | Tashiro | ^33^ |
| Yugo |  | Kawai | ^33^ |
| Yoshitaka |  | Nakata | ^3^ |
| Hiroki |  | Futatsusako | ^3^ |
| Ayaka |  | Sakamoto | ^3^ |
| Naoko |  | Yasuhara | ^3^ |
| Takao |  | Hashiguchi | ^33^ |
| Tateki |  | Suzuki | ^33^ |
| Kanako |  | Kimura | ^33^ |
| Jiei |  | Sasaki | ^33^ |
| Yukari |  | Nakajima | ^33^ |
| Hisano |  | Yajima | ^33^ |
| Takashi |  | Irie | ^34^ |
| Ryoko |  | Kawabata | ^34^ |
| Kaori |  | Sasaki-Tabata | ^35^ |
| Ryo |  | Shimizu | ^10^ |
| MST | Monira | Begum | ^10^ |
| Michael |  | Jonathan | ^10^ |
| Yuka |  | Mugita | ^10^ |
| Sharee |  | Leong | ^10^ |
| Otowa |  | Takahashi | ^10^ |
| Kimiko |  | Ichihara | ^10^ |
| Takamasa |  | Ueno | ^36^ |
| Chihiro |  | Motozono | ^36^ |
| Mako |  | Toyoda | ^36^ |
| Akatsuki |  | Saito | ^37^ |
| Anon |  | Kosaka | ^37^ |
| Miki |  | Kawano | ^37^ |
| Natsumi |  | Matsubara | ^37^ |
| Tomoko |  | Nishiuchi | ^37^ |
| Jiri |  | Zahradnik | ^38^ |
| Prokopios |  | Andrikopoulos | ^38^ |
| Miguel |  | Padilla-Blanco | ^38^ |
| Aditi |  | Konar | ^38^ |

**【Affiliation】** *The numbers are consistent with the main text.

^1^ Division of Systems Virology, Department of Microbiology and Immunology, The Institute of Medical Science, The University of Tokyo, Tokyo, Japan

^3^ Center for iPS Cell Research and Application (CiRA), Kyoto University, Kyoto, Japan

^4^ Department of Microbiology and Immunology, Faculty of Medicine, Hokkaido University, Sapporo, Japan.

^8^ Department of Cancer Pathology, Faculty of Medicine, Hokkaido University, Sapporo, Japan

^10^ Division of Molecular Virology and Genetics, Joint Research Center for Human Retrovirus infection, Kumamoto University, Kumamoto, Japan

^13^ Division of Risk Analysis and Management, International Institute for Zoonosis Control, Hokkaido University, Sapporo, Japan

^21^ Tokyo Metropolitan Institute of Public Health, Tokyo, Japan

^31^ Hokkaido University, Sapporo, Japan

^32^ Tokai University School of Medicine, Isehara, Japan

^33^ Kyoto University, Kyoto, Japan

^34^ Hiroshima University, Hiroshima, Japan

^35^ Kyushu University, Fukuoka, Japan

^36^ Kumamoto University, Kumamoto, Japan

^37^ Miyazaki University, Miyazaki, Japan

^38^ Charles University, Vestec-Prague, Czechia
